# TGF-β-induced hepatocyte lincRNA-p21 contributes to liver fibrosis in mice

**DOI:** 10.1038/s41598-017-03175-0

**Published:** 2017-06-07

**Authors:** Xiaolong Tu, Yuanyuan Zhang, Xiuxiu Zheng, Jia Deng, Huanan Li, Zhiqian Kang, Zhipeng Cao, Zhen Huang, Zhi Ding, Lei Dong, Jiangning Chen, Yuhui Zang, Junfeng Zhang

**Affiliations:** 1 State Key Laboratory of Pharmaceutical Biotechnology, School of Life Science, Nanjing University, Nanjing, 210093 P.R. China; 2 Jiangsu Engineering Research Center for microRNA Biology and Biotechnology, Nanjing, 210093 P.R. China

## Abstract

Hepatocyte death, as well as the following inflammatory and fibrogenic signaling cascades, is the key trigger of liver fibrosis. Here, we isolated hepatocytes from CCl_4_-induced fibrotic liver and found that hepatocyte lincRNA-p21 significantly increased during liver fibrosis. The increase of hepatocyte lincRNA-p21 was associated with the loss of miR-30, which can inhibit TGF-β signaling by targeting KLF11. We revealed that lincRNA-p21 modulated miR-30 availability by acting as a competing endogenous RNA (ceRNA). The physiological significance of this interaction is highlighted by the feedback loop, in which lincRNA-p21 works as a downstream effector of the TGF-β signaling to strengthen TGF-β signaling and mediate its role in promoting liver fibrosis by interacting with miR-30. *In vivo* results showed that knockdown of hepatocyte lincRNA-p21 greatly reduced CCl_4_-induced liver fibrosis and inflammation, whereas ectopic expression of miR-30 in hepatocyte exhibited the similar results. Mechanistic studies further revealed that inhibition of miR-30 impaired the effects of lincRNA-p21 on liver fibrosis. Additionally, lincRNA-p21 promoted hepatocyte apoptosis *in vitro* and *in vivo*, whereas the proliferation rate of hepatocyte was suppressed by lincRNA-p21. The pleiotropic roles of hepatocyte lincRNA-p21 suggest that it may represent an unknown paradigm in liver fibrosis and serve as a potential target for therapy.

## Introduction

Hepatic fibrosis occurs during most chronic liver diseases and is characterized by the excessive production of extracellular matrix (ECM)^1, 2^. Following continued liver injury, quiescent hepatic stellate cells (HSCs) are exposed to apoptotic hepatocytes, inflammatory and profibrogenic factors, and transdifferentiate into myofibroblasts that synthesize large amounts of ECM proteins^3^. Despite the fact that HSCs play a pivotal role in hepatic fibrogenesis, hepatocytes are the dominant cell type residing in the liver and actively orchestrate the profibrogenic responses. In different animal models, as well as various liver diseases, development of liver fibrosis is associated with significant hepatocyte injury. Increased hepatocyte death promotes inflammatory cell recruitment, and subsequently triggers activation of inflammatory and fibrogenic signaling cascades^4^. There is ample evidence that apoptotic hepatocytes promote the secretion of pro-inflammatory and profibrogenic cytokines and trigger HSC activation^5, 6^. Hepatocyte-specific deletion of Bcl-xl, Mcl-1 or TAK1 leads to selective increase of hepatocyte apoptosis and triggers liver fibrosis, thus providing a direct link between hepatocyte apoptosis and fibrogenesis^7–9^. Among the profibrogenic cytokines released by hepatocyte, TGF-β plays pleiotropic roles in the progression of liver fibrosis. Enhanced TGF-β signaling has been implicated during hepatic fibrosis in different animal models and human patients^10^. In hepatocyte, TGF-β signaling promotes cell damage, profibrogenic and inflammatory cytokine production, leads to cellular apoptosis and growth inhibition, and thus contributes to hepatic fibrogenesis^11, 12^.

Long noncoding RNAs (lncRNAs) are defined as greater than 200 nt and unable to be translated into proteins. Accumulating data show that lncRNAs are involved in regulating gene expression through various mechanisms and participate in the regulation of a variety of cellular events^13, 14^. So far, the deregulations of lncRNA have been shown in various disease states. Kinds of lncRNAs are involved in liver regeneration, neoplasia and other liver diseases^15–17^. However, the involvement of lncRNA in liver fibrogenesis, especially the deregulation of lncRNA in hepatocyte upon liver injury, remains elusive. Here, we isolated hepatocytes from carbon tetrachloride (CCl_4_)-induced fibrotic liver and found that hepatocyte lincRNA-p21 (long intervening noncoding RNA-p21) significantly increased during liver fibrosis. LincRNA-p21 was initially identified as a transcriptional target of p53, and was characterized to mediate p53-dependent apoptosis but not cell cycle arrest in doxorubicin-treated mouse embryo fibroblasts^18, 19^. However, there are conflicting reports on the role of lincRNA-p21 in apoptosis^20–22^.

The MicroRNA (miRNA) profiling of fibrotic liver has identified a panel of deregulated miRNAs that contributes to liver fibrogenesis^23, 24^. LncRNA transcripts can regulate microRNA activity by acting as either competitive endogenous RNAs (ceRNA) or as microRNA sponges, thereby alleviating the inhibitory effect of microRNAs on their respective mRNA targets^25–27^. In the present study, we find that hepatocyte lincRNA-p21 can function as a ceRNA by binding miR-30, and therefore participating in the regulation of TGF-β signaling and liver fibrosis. Moreover, our results reveal that lincRNA-p21 contributes to promoting hepatocyte apoptosis and suppressing hepatocyte growth in the fibrotic liver. Knockdown of hepatocyte lincRNA-p21 *in vivo* blunted CCl_4_-induced hepatocyte apoptosis and therefore reduced the infiltration of inflammatory cells and the secretion of pro-inflammatory and profibrogenic cytokines in the CCl_4_-induced fibrotic liver.

## Results

### LincRNA-p21 is upregulated in the hepatocyte during liver fibrosis

**Figure 1:**
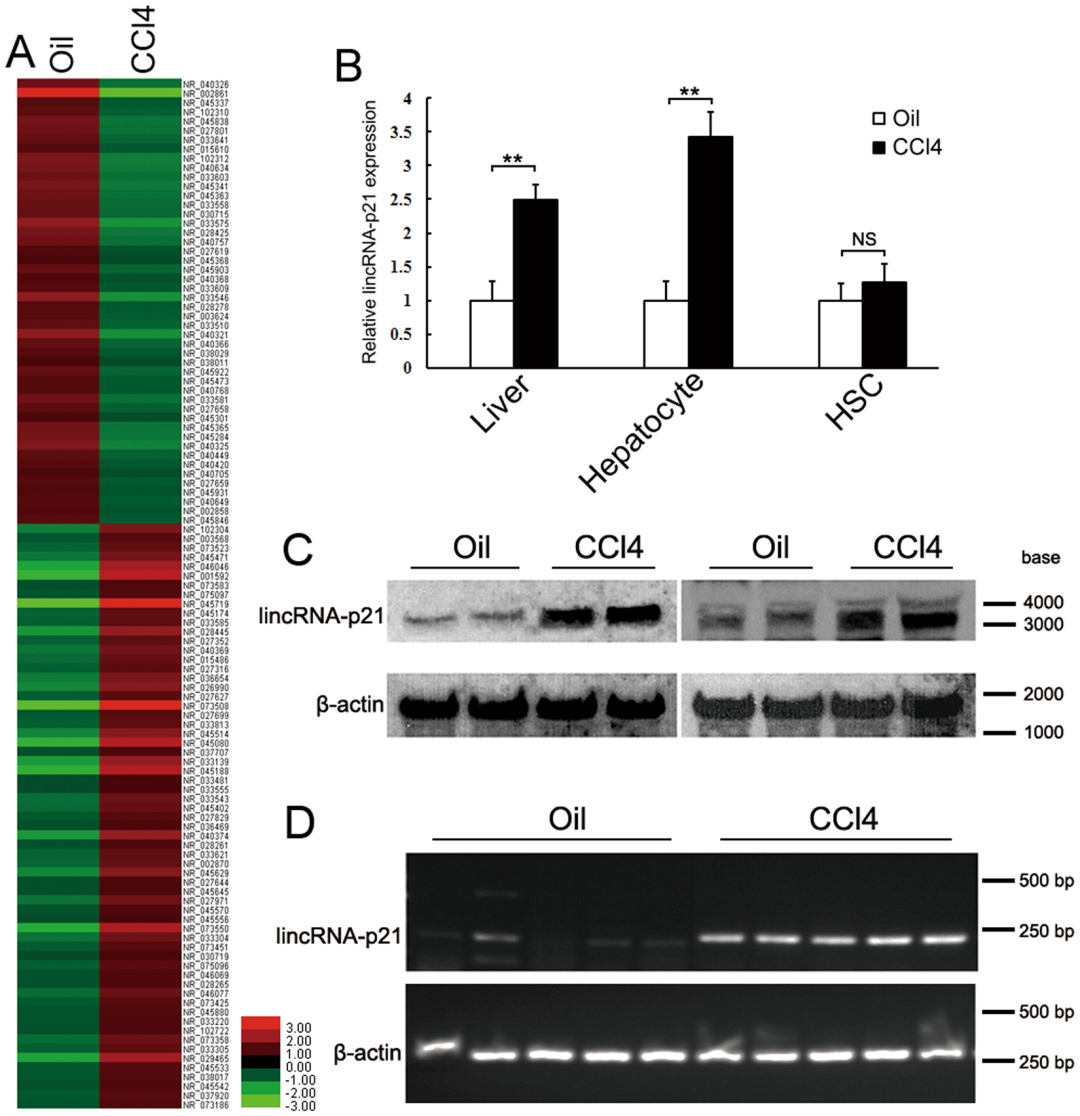
LincRNA-p21 is upregulated in the hepatocyte of CCl_4_-induced fibrotic liver. (**A**) Geometric mean-centered, hierarchical cluster heat map from RNA-seq. 111 annotated lncRNAs (p < 0.05) were represented in the hepatocytes isolated from CCl_4_-induced fibrotic liver (n = 5) compared with hepatocytes from oil-treated liver (n = 5). (**B**) LincRNA-p21 expression was determined in CCl_4_-induced fibrotic liver, hepatocyte and HSC by qRT-PCR. The relative gene expression was normalized against β-actin unless noted otherwise in this study. The results are shown as fold change compared with oil- treated group. (**C** and **D**) Hepatocyte lincRNA-p21 was detected by northern blot or RT-PCR. RNAs were extracted from isolated hepatocytes of CCl_4_-induced fibrotic liver. Data are the mean ± SEM of three independent experiments. **P < 0.01. NS, no significant change. The original northern blot and gel images are shown in Supplementary Figure S9.

As a first attempt to investigate the deregulation of hepatocyte lncRNAs during liver fibrogenesis, we isolated hepatocytes from 5 CCl_4_-induced fibrotic liver and 5 oil-treated sham liver. Total RNA were prepared and used to conduct transcriptome RNA sequencing (RNA-seq). Basing on an absolute fold change cutoff value of 1 in log2 scale, we identified 111 aberrantly expressed lncRNA transcripts (63 upregulated and 48 downregulated) in the hepatocytes from fibrotic liver compared with those from oil-treated liver (Fig. 1A). Among the highly expressed lncRNAs, we focused on the intergenic lncRNA-p21, which was initially identified as a transcriptional target of p53.

To confirm the RNA-seq result, we determined lincRNA-p21 expression in the isolated hepatocyte by qRT-PCR, showing that hepatocyte lincRNA-p21 significantly increased after CCl_4_-injection (Fig. 1B). We next examined the lincRNA-p21 expression in the fibrotic liver and isolated HSCs and found that hepatic lincRNA-p21 increased after CCl_4_-treatment. However, the increase in the HSCs wasn’t significant (Fig. 1B). Northern blot and Semi-qRT-PCR results further confirmed the upregulation of lincRNA-p21 in hepatocyte during liver fibrosis (Fig. 1C and D).

### LincRNA-p21 is physically associated with the miR-30

**Figure 2:**
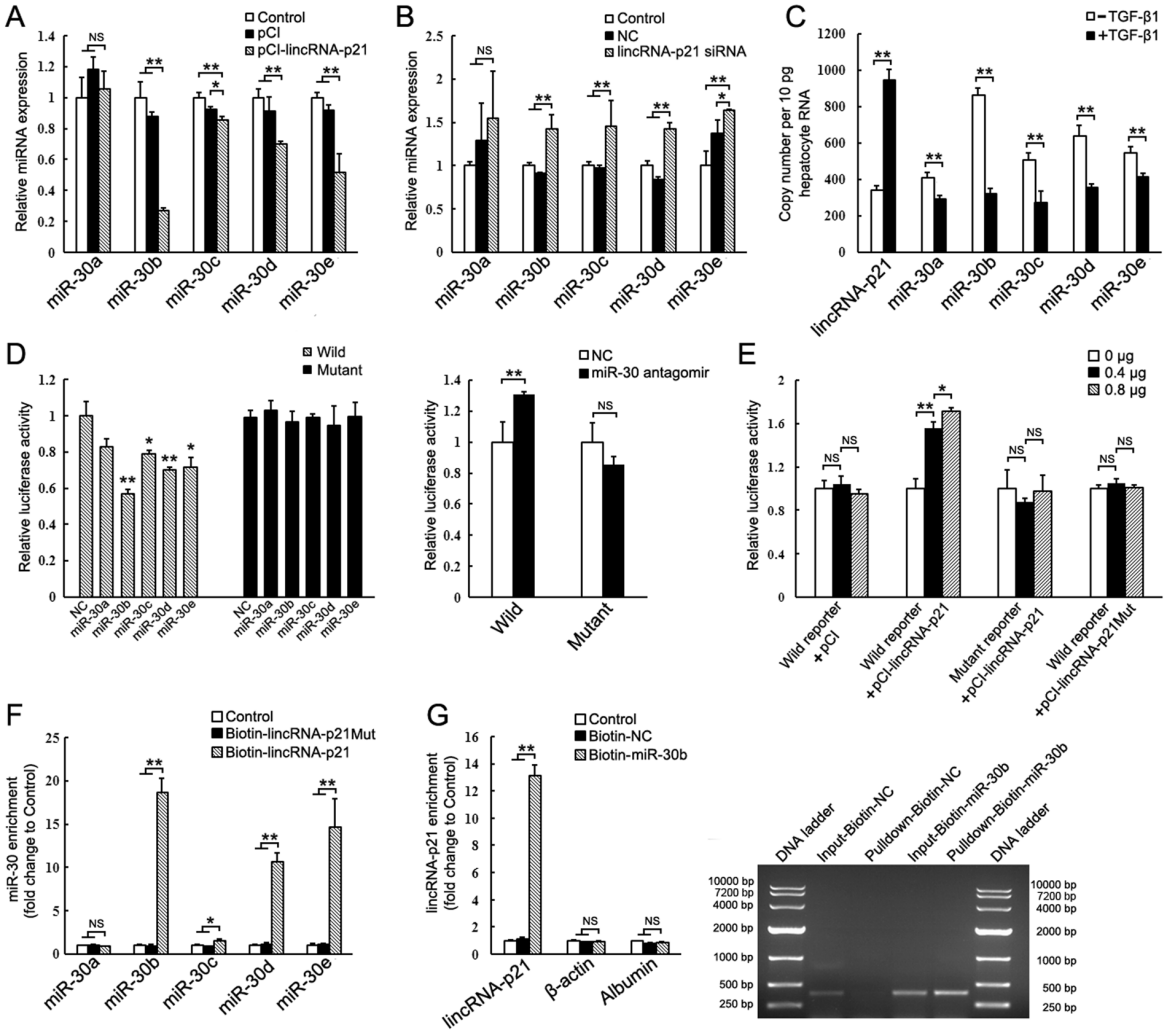
LincRNA-p21 interacts with miR-30. (**A**) Relative expression of miR-30 in AML12 cells transfected with pCI-lincRNA-p21 for 24 h. (**B**) Relative expression of miR-30 in AML12 cells transfected with lincRNA-p21 siRNA for 24 h. miR-30 levels were normalized against U6 snRNA unless noted otherwise in this study. The results are shown as fold change compared with control. (**C**) The copy numbers of lincRNA-p21 and miR-30 in AML12 cells treated with or without TGF-β1 were quantified. (**D**) Analysis of luciferase activity in AML12 cells cotransfected with indicated luciferase reporters containing wild or mutant lincRNA-p21 cDNA, and miR-30 mimics or miR-30 antagomir. (**E**) Wild type or mutant luciferase reporter was transfected into AML12 cells, together with 0, 0.4 or 0.8 μg of pCI-lincRNA-p21 or pCI-lincRNA-p21Mut. (**F**) miR-30 is associated with lincRNA-p21. AML12 cells were harvested and mixed with biotinylated lincRNA-p21 (Biotin-lincRNA-p21) or biotinylated lincRNA-p21 Mutant (Biotin- lincRNA-p21Mut) to perform biotin-based pull down. miR-30 enrichment was determined by qRT-PCR and normalized to control. (**G**) lincRNA-p21 is associated with miR-30. AML12 cells were transfected with biotinylated miR-30b (Biotin-miR-30b) or its biotinylated mimic control (Biotin-NC) for 24 h. Cells were then harvested for biotin-based pull-down. After washing and enrichment of beads/RNA complex, RNA was eluted from the streptavidin beads. Left, lincRNA-p21 enrichment was determined by qRT-PCR and normalized to control without any transfection. Right, Eluted RNA was amplified by RT-PCR with oligonucleotides spanning region of lincRNA-p21 and run on 1% agarose gel. Total inputs (Input-Biotin-NC and Input-Biotin-miR-30b) are indicated as total RNA isolated from Biotin-NC or Biotin-miR-30b-transfected AML12 cells. The results are shown as fold change compared with control group. Data are the mean ± SEM of three independent experiments. *P < 0.05, **P < 0.01. NS, no significant change.

LncRNAs can function as ceRNA by competitively binding microRNAs^28–30^. A set of putative miRNA binding sites located on lincRNA-p21 were identified by TargetScan algorithm (http://www.targetscan.org/). Among them, RNAhybrid analysis (http://bibiserv.techfak.uni-bielefeld.de/rnahybrid/) further revealed a healthy minimum free energy of hybridization between lincRNA-p21 and miR-30 family members (Supplementary Figure S1A). To examine the interactions between lincRNA-p21 and miR-30, the nontumorigenic mouse hepatocyte cell line AML12 were transiently transfected with the expression plasmid pCI-lincRNA-p21 that contains the murine lincRNA-p21 cDNA. Overexpression of lincRNA-p21 (Supplementary Figure S1B) significantly reduced miR-30b, -30c, -30d and -30e levels (Fig. 2A). Conversely, knockdown of lincRNA-p21 by siRNA exerted the opposite effects (Fig. 2B). However, transfection of miR-30s into AML12 cells didn’t significantly affected lincRNA-p21 level (Supplementary Figure S1C).

Using the standard curve method, we measured the absolute copy numbers of lincRNA-p21 and miR-30s in AML12 treated with or without TGF-β1, showing that the abundance of lincRNA-p21 and miR-30 are comparable (Fig. 2C). Thus, lincRNA-p21 may be able to function as a ceRNA for miR-30. To confirm the interaction between lincRNA-p21 and miR-30, we inserted the lincRNA-p21 cDNA downstream of the firefly luciferase reporter gene. Transfection of miR-30 greatly decreased the luciferase activity of the wild type reporter with normal binding sites for miR-30, but not that with the mutant binding sites. In contrast, miR-30 antagomir inhibited endogenous miR-30 and increased the luciferase activity (Fig. 2D). Next, we transfected the luciferase reporter into AML12 cells together with increasing amounts of pCI-lincRNA-p21. The luciferase activity increased in response to pCI-lincRNA-p21 in a dose-dependent manner, suggesting that ectopically expressed lincRNA-p21 sequestered endogenous miR-30 and prevented it from suppressing luciferase expression (Fig. 2E). Meanwhile, pCI-lincRNA-p21Mut, in which the predicted miR-30 binding site was mutated, failed to increase the luciferase activity (Fig. 2E).

Finally, we performed biotin–avidin pull-down assay using *in vitro*-synthesized biotinylated lincRNAp21 or lincRNAp21 mutant harboring mutated miR-30 binding site. miR-30s were significantly enriched in the RNA immunoprecipitation (RIP) pulled down by biotinylated lincRNA-p21, but not by biotinylated lincRNAp21 mutant (Fig. 2F). Meanwhile, other miRNAs (including miR-122, -18, -101a, -101b) that exhibit similar or higher expression levels didn’t obviously enrich in the RIP (Supplementary Figure S1D). The specific association between miR-30 and lincRNA-p21 was also validated by affinity pull-down of miR-30. AML12 cells were transfected with biotinylated miR-30b, then harvested for biotin-based pull-down assay. RT-PCR and qRT-PCR results confirmed that lincRNA-p21 was specially pulldown by biotinylated miR-30b (Fig. 2G).

### Hepatocyte miR-30 inhibits liver fibrosis

**Figure 3:**
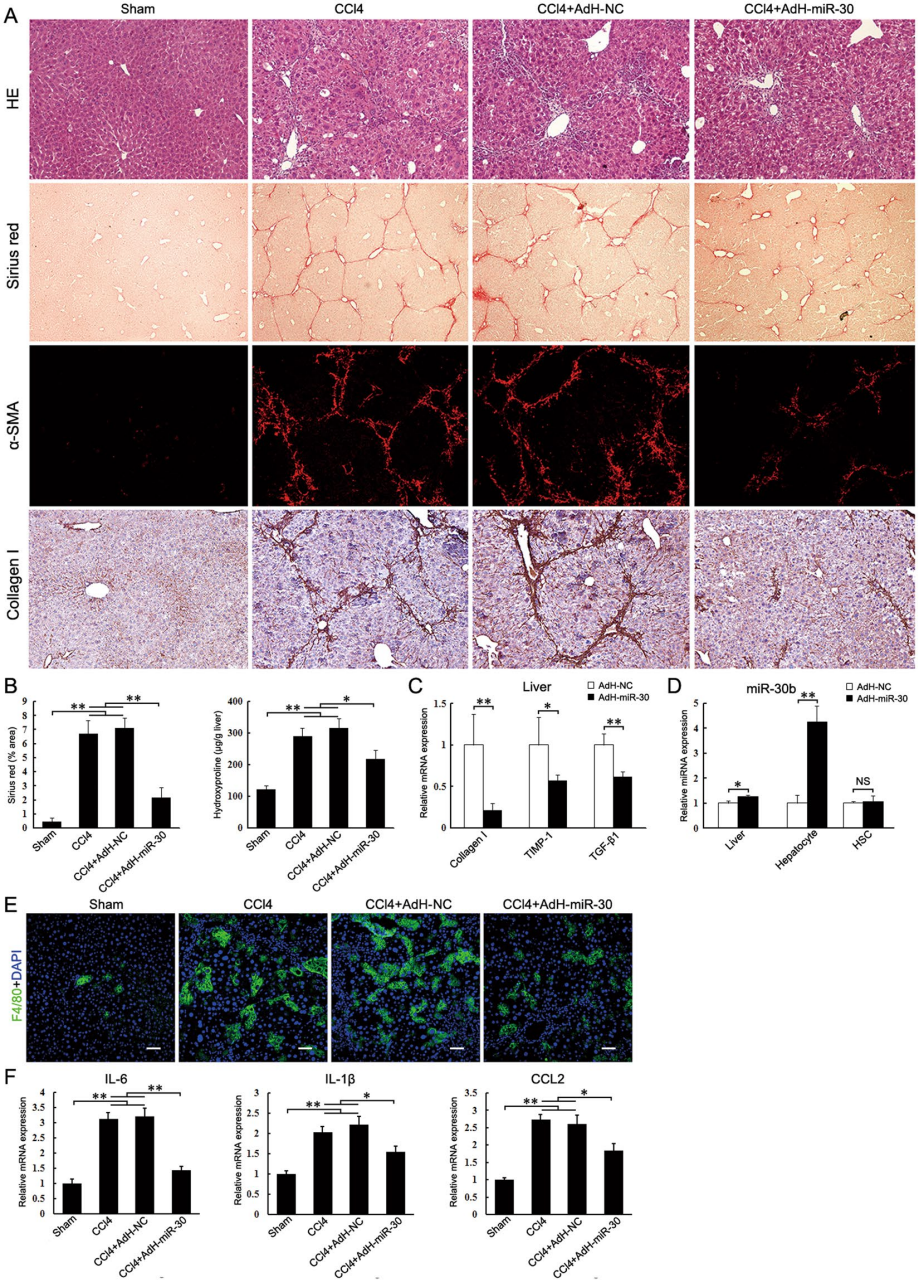
Ectopic expression of miR-30b in hepatocyte suppresses CCl_4_-induced liver fibrosis. Mice were treated with oil (Sham, n = 6), CCl_4_ (CCl4, n = 6), CCl_4_ in combination with injection of AdH-NC (CCl4 + AdH-NC, n = 6) and CCl_4_ in combination with injection of AdH-miR-30 (CCl4 + AdH-miR-30, n = 6). The mice were sacrificed after 3 weeks CCl_4_ treatment. (**A**) Liver fibrosis was evaluated by H&E staining (100×), sirius red staining (40×), α-SMA immunofluorescence staining (100×) and collagen I immunohistochemical staining (100×). (**B**) Quantifications of the sirius red positive area and hepatic hydroxyproline content. The hydroxyproline contents are expressed as μg/g wet liver weight. (**C**) Hepatic Collagen I, TIMP-1 and TGF-β1 mRNA were determined by qRT-PCR. (**D**) miR-30b level in the whole liver, isolated hepatocytes and HSCs were determined by qRT-PCR respectively. (**E**) Immunofluorescence staining of liver sections with anti-F4/80 antibody. Scale bar, 50 μm. (**F**) Hepatic IL-6, IL-1β and CCL2 mRNA were determined by qRT-PCR. The results are shown as fold change compared with Sham group or AdH-NC group mice. Data are the mean ± SEM of three independent experiments. *P < 0.05, **P < 0.01. NS, no significant change.

We previously found that hepatic miR-30s decreased in the fibrotic liver and HSC-specific upregulation of miR-30 prevented liver fibrosis^31^. Here, we examined miR-30s expressions in the hepatocyte after CCl_4_ treatment and found that they decreased about 1~4-fold (Supplementary Figure S2A). Thus, we hypothesized that hepatocyte lincRNA-p21 and miR-30 are inversely associated and involved in liver fibrosis. To test this, we constructed adenovirus AdH-miR-30 and AdH-NC that can specifically express miR-30b or control in hepatocyte *in vivo* under the control of albumin promoter. AdH-miR-30 could significant increased miR-30b expression in AML12, but not in the cultured HSC cell line HSC-T6 (Supplementary Figure S2B). Two days before the first injection of CCl_4_, AdH-miR-30 or AdH-NC was injected into mice via tail vein. Ectopic expression of miR-30 greatly inhibited CCl_4_-induced liver fibrosis as observed by histological examination (Fig. 3A), and significantly decreased collagen deposition and hepatic hydroxyproline level (Fig. 3B). Notably, TGF-β1, Col1a1 and tissue inhibitor of metalloproteinase-1 (TIMP-1) were also greatly reduced in the AdH-miR-30-injected mice. Administration of AdH-miR-30 led to miR-30b increase in the liver tissue (Fig. 3C). Moreover, miR-30b expression increased in the hepatocytes of AdH-miR-30-injected mice, but not in the HSCs (Fig. 3D).

Liver inflammation, triggered by injured hepatocytes, is one of the most characteristic features of chronic liver injury and associated with the development of fibrosis. CCl_4_ administration induced significant inflammatory cell infiltration in liver. Notably, increased infiltration of macrophages was limited in AdH-miR-30 group mice (Fig. 3E). Consistent with the histology results, hepatic expression of inflammatory genes, including interleukin-6 (IL-6), chemokine ligand 2 (CCL2) and IL-1β, were suppressed in AdH-miR-30 group (Fig. 3F).

### Knockdown of lincRNA-p21 in hepatocyte prevents liver fibrosis

**Figure 4:**
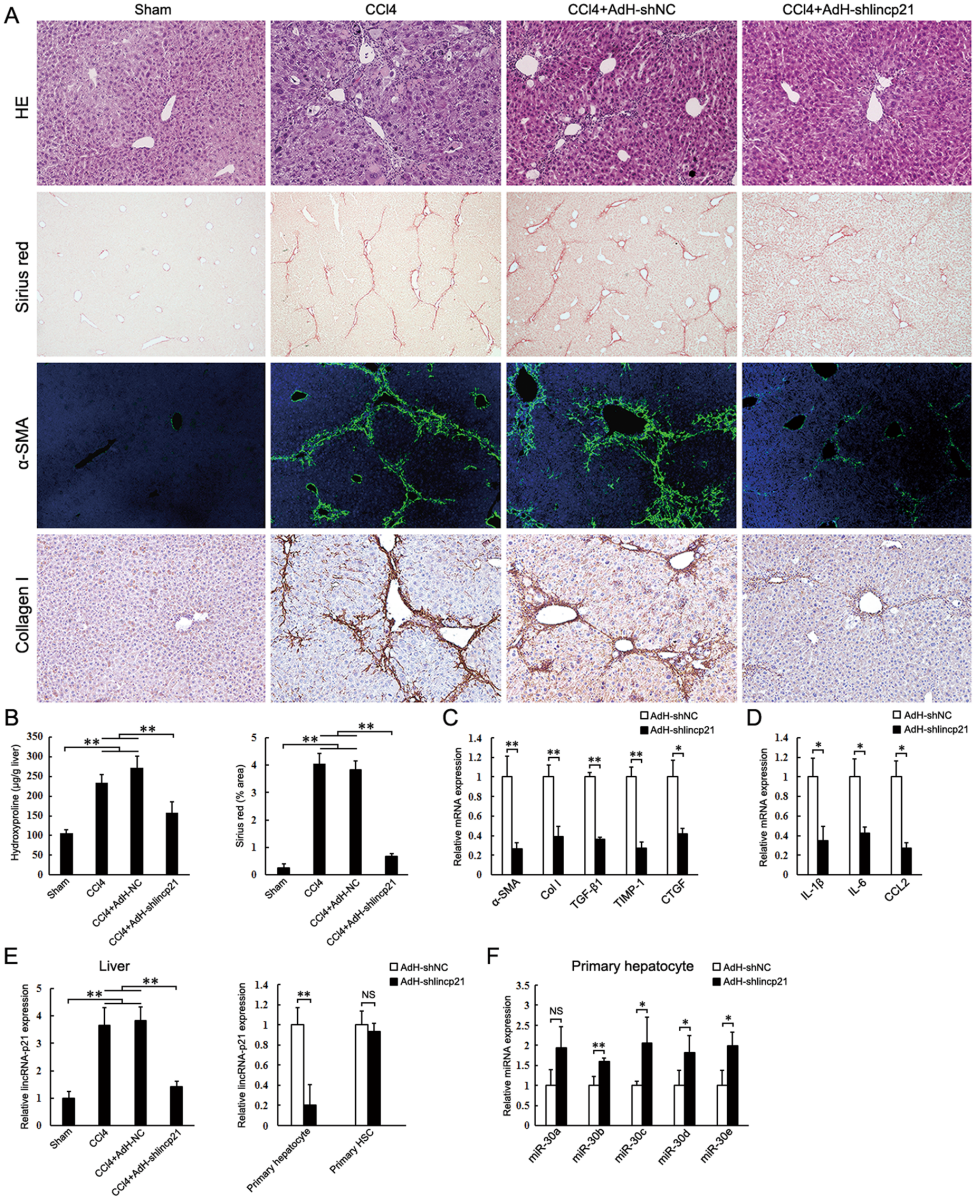
Knockdown of lincRNA-p21 in hepatocyte suppresses CCl_4_-induced liver fibrosis. Mice were treated with oil (Sham, n = 6), CCl_4_ (CCl4, n = 6), CCl_4_ in combination with injection of AdH-shNC (CCl4 + AdH-shNC, n = 6) and CCl_4_ in combination with injection of AdH-shlincp21 (CCl4 + AdH-shlincp21, n = 6). The mice were sacrificed after 3 weeks CCl_4_ treatment. (**A**) Liver fibrosis was evaluated by H&E staining (100×), sirius red staining (40×), α-SMA immunofluorescence staining (100×) and collagen I immunohistochemical staining (100×). (**B**) Quantifications of the sirius red positive area and hepatic hydroxyproline content. The hydroxyproline contents are expressed as μg/g wet liver weight. (**C**) Hepatic α-SMA, collagen I, TGF-β1, TIMP-1 and CTGF mRNA were determined by qRT-PCR. (**D**) Hepatic IL-6, IL-1β and CCL2 mRNA were determined by qRT-PCR. (**E**) lincRNA-p21 levels in the whole liver, hepatocytes and HSCs were determined by qRT-PCR respectively. (**F**) miR-30 levels in the isolated hepatocytes were determined by qRT-PCR. The results are shown as fold change compared with Sham group or AdH-shNC group mice. Data are the mean ± SEM of three independent experiments. *P < 0.05, **P < 0.01. NS, no significant change.

We next investigated the role of hepatocyte lincRNAp21 in liver fibrosis. Mice were injected in the tail vein before the first injection of CCl_4_ with the adenovirus AdH-shlincp21 or AdH-NC, which can express lincRNA-p21 shRNA or control under the control of albumin promoter (Supplementary Figure S3A). Compared to the AdH-shNC-infected mice, AdH-shlincp21-infected mice showed a marked reduction of hepatic fibrosis as observed by histological examination (Fig. 4A), and significantly decreased collagen deposition and hepatic hydroxyproline level (Fig. 4B). LincRNA-p21 silencing also significantly reduced the expression of α-SMA, Col1a1, TGF-β1, TIMP-1 and CTGF (Fig. 4C). Additionally, administration of AdH-shlincp21 reduced the infiltration of hepatic macrophages (Supplementary Figure S3B) and the upregulation of hepatic IL-6, IL-1 and CCL2 in CCl_4_-treated mice (Fig. 4D).

Administration of AdH-shlincp21 reduced hepatic lincRNA-p21 (Fig. 4E). We then isolated hepatocyte and HSC from the fibrotic liver. LincRNA-p21 greatly decreased in the hepatocytes from AdH-shlincp21 group, but not in the isolated HSCs (Fig. 4E). Moreover, the miR-30s in the isolated hepatocytes from AdH-shlincp21 group mice significantly increased, suggesting that AdH-shlincp21 might prevent liver fibrosis by increasing miR-30 in hepatocyte (Fig. 4F).

### Hepatocyte lincRNA-p21 regulates liver fibrosis through interacting with miR-30

**Figure 5:**
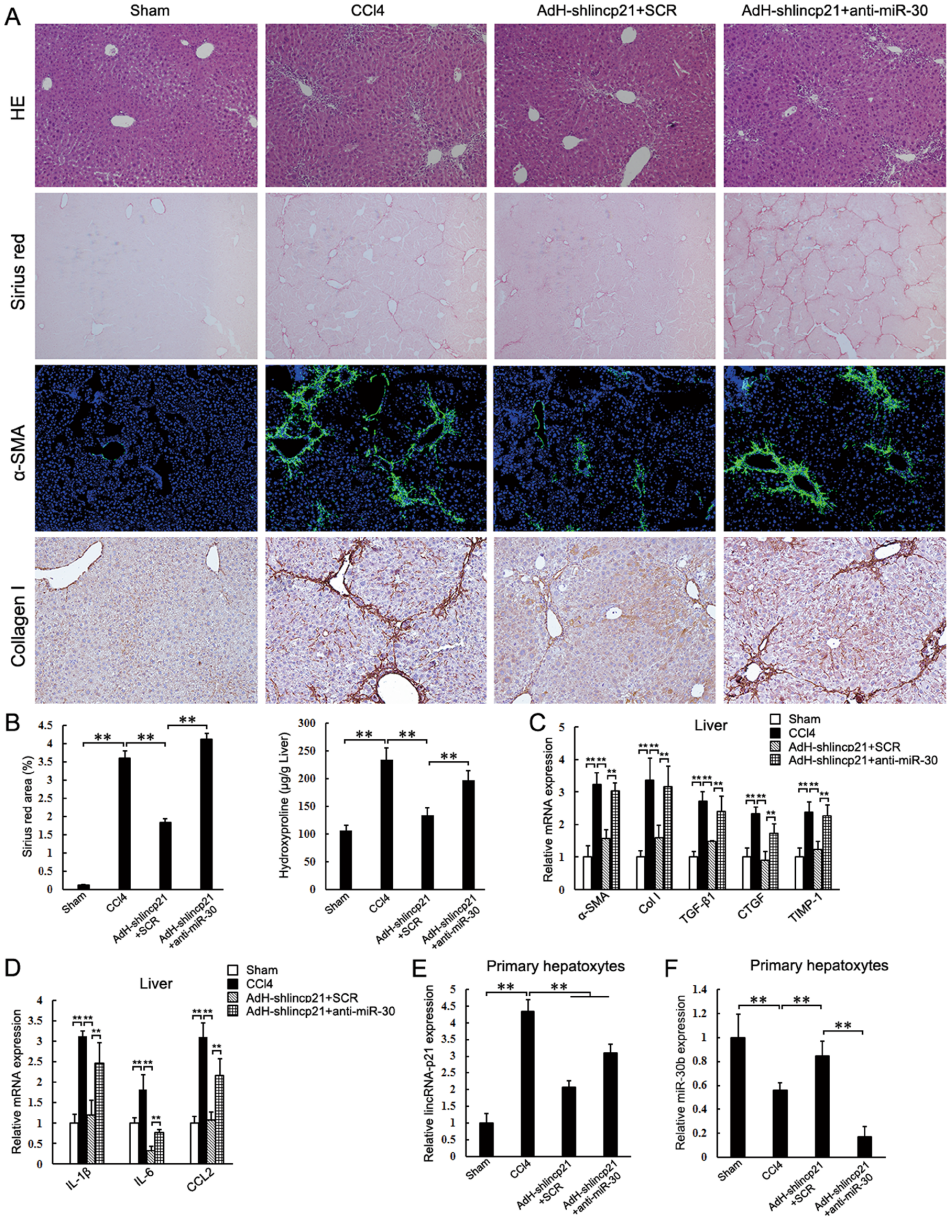
Inhibition of miR-30 impairs the effects of lincRNA-p21 knockdown on CCl_4_-induced liver fibrosis. Mice were treated with oil (Sham, n = 6), CCl_4_ (CCl4, n = 6), CCl_4_ in combination with injection of AdH-shlincp21 and SCR (AdH-shlincp21 + SCR, n = 6) and CCl_4_ in combination with injection of AdH-shlincp21 and anti-miR-30 (AdH-shlincp21 + anti-miR-30, n = 6). The mice were sacrificed after 3 weeks CCl_4_ treatment. (**A**) Liver fibrosis was evaluated by H&E staining (100×), sirius red staining (40×), α-SMA immunofluorescence staining (100×) and collagen I immunohistochemical staining (100×). (**B**) Quantifications of the sirius red positive area and hepatic hydroxyproline content. The hydroxyproline contents are expressed as μg/g wet liver weight. (**C**) Hepatic α-SMA, collagen I, TGF-β1, TIMP-1 and CTGF mRNA were determined by qRT-PCR. (**D**) Hepatic IL-6, IL-1β and CCL2 mRNA were determined by qRT-PCR. (**E** and **F**) lincRNA-p21 and miR-30b levels in the isolated hepatocytes were determined by qRT-PCR respectively. The results are shown as fold change compared with Sham group mice. Data are the mean ± SEM of three independent experiments. *P < 0.05, **P < 0.01.

We reasoned that, if hepatocyte lincRNA-p21 regulates liver fibrosis by interacting with miR-30, inhibition of miR-30 would show inhibitory effects on the protective function of AdH-shlincp21 in liver fibrosis. Inhibition of miRNAs with modified antisense oligo-ribonucleotides has been proven to be efficient in silencing endogenous miRNAs in multi tissues^32, 33^. To test our hypothesis, anti-miR-30, a phosphorothioate-modified antisense oligonucleotides specific for miR-30, and scrambled control (SCR), were intravenously injected into CCl_4_-treated mice weekly during the liver fibrosis development. AdH-shlincp21 was injected in the tail vein two days before the first treatment of CCl_4._ In SCR-injected group, AdH-shlincp21 significantly inhibited liver fibrosis. However, in anti-miR-30 group, AdH-shlincp21 failed to exert the inhibitory effects (Fig. 5A and B). The expression of hepatic profibrogenic markers (α-SMA, Col1a1, TGF-β1, CTGF and TIMP-1) also significantly increased in anti-miR-30 group (Fig. 5C). In addition, hepatic expression of IL-6, IL-1 and CCL2 significantly increased in anti-miR-30b group mice (Fig. 5D).

AdH-shlincp21 prevented the increase of hepatocyte lincRNA-p21 and led to a significant increase of miR-30b in the isolated hepatocytes from fibrotic liver (Fig. 5E). However, the injection of miR-30 antisense oligonucleotides decreased miR-30b in the hepatocyte (Fig. 5F). Collectively, our results suggest that hepatocyte lincRNA-p21 contributes to liver fibrosis by interacting with miR-30.

### LincRNA-p21 is induced by TGF-β in the hepatocyte

**Figure 6:**
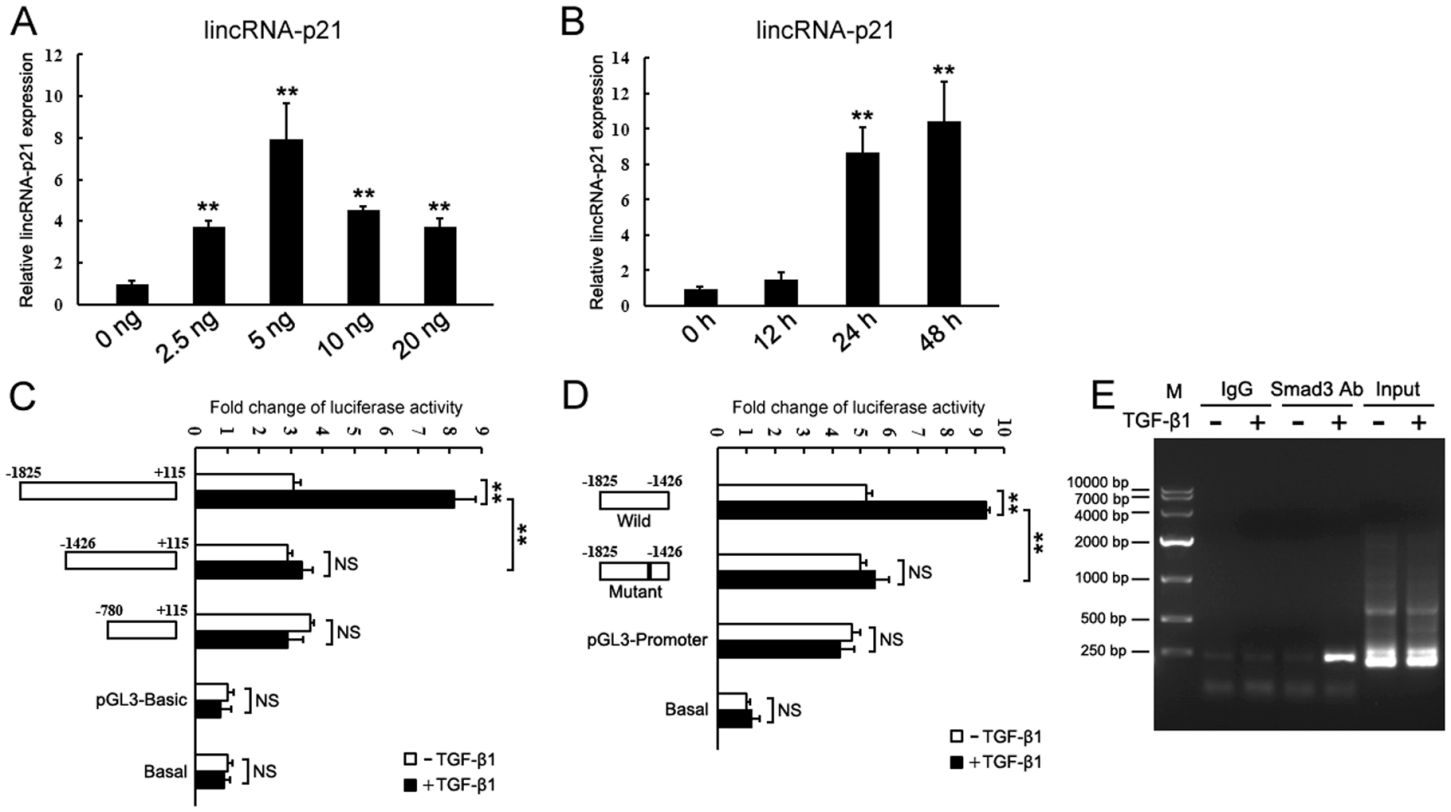
LincRNA-p21 is induced by TGF-β. (**A**) The expression of lincRNA-p21 in AML12 cell treated by TGF-β1 at indicated concentrations for 24 h. (**B**) The expression of lincRNA-p21 in AML12 cell treated by TGF-β1 at a final concentration of 5 ng/μl for indicated times. (**C** and **D**) Schematic representation of lincRNA-p21 promoter reporter constructs using pGL3-Basic (**C**) or pGL3-Promoter (**D**) and analysis of their reporter activities after treatment with TGF-β1 for 24 h. The results are shown as fold change compared with cells without plasmid transfection. (**E**) ChIP assays for Smad3 were performed with chromatin from AML12 cells treated with TGF-β1 for 24 h. Precipitated DNA was amplified with oligonucleotides spanning regions of predicted Smad3 binding site. Total inputs are indicated. Data are the mean + SEM of three independent experiments. **P < 0.01. NS, no significant change.

Given the prominent role of TGF-β/Smad signaling in hepatocyte death and hepatic fibrogenesis, we investigated the regulation of hepatocyte lincRNA-p21 in response to TGF-β1. We found that lincRNA-p21 was induced by TGF-β1 in a dose- and time-dependent manner (Fig. 6A and B). The inhibitor of type I TGF-β receptor kinase SB431542 completely blocked the increase of TGF-β-induced lincRNA-p21 (Supplementary Figure S4A). Since lincRNA-p21 was initially identified as a transcriptional target of p53, we examined the expression of p53 in TGF-β1-treated AML12 cells. We found that TGF-β1 didn’t increase p53 production, and thus excluded the possibility that TGF-β1 increased lincRNA-p21 expression via p53 (Supplementary Figure S4B).

We searched for potential Smad-binding sites in the upstream region of lincRNA-p21 using rVista 2.0 (http://rvista.dcode.org/), and found 3 conserved binding sites at −1520 bp, −944 bp and −530 bp upstream of lincRNA-p21 in mouse genome. The predicted murine lincRNA-p21 promoter includes the 1400 bp upstream and 200 bp downstream of the first exon^34^. To identify the *cis*-element responsible for TGF-β induction, we constructed a set of pGL3-Basic luciferase reporters carrying various genomic sequences spanning from −1,825 to + 115 (relative to transcription start site), and subjected them to luciferase assays. The construct encompassing −1825 to +115 bp had a higher promoter activity in response to TGF-β1 (Fig. 6C). We then cloned the −1825 bp to −1426 bp fragment into pGL3-Promoter luciferase reporter, showing that the this fragment contained the TGF-β-response element (Fig. 6D). Finally, we mutated the predicted Smad-binding sites (from −1520 to −1512 bp) and found the mutation abolished the TGF-β-induced luciferase activities (Fig. 6D). Chromatin immunoprecipitation (ChIP) also demonstrated that the antibody against Smad3 could immunoprecipitate the predicted DNA fragments from TGF-β1-treated hepatocyte (Fig. 6E).

Notably, we have previously reported that TGF-β1 reduced miR-30 in hepatocyte^35^. To ascertain the underlying mechanism responsible for miR-30 decrease in response to TGFβ, we determined the expression of pri-miR-30s in TGFβ-treated AML12 cells, showing that TGFβ didn’t obviously suppress the transcription of pri-miR-30s (Supplementary Figure S4C). Thus, TGFβ-induced lincRNA-p21 might be responsible for the decrease of miR-30.

### LincRNA-p21 activates TGF-β/Smad signaling in the hepatocyte

**Figure 7:**
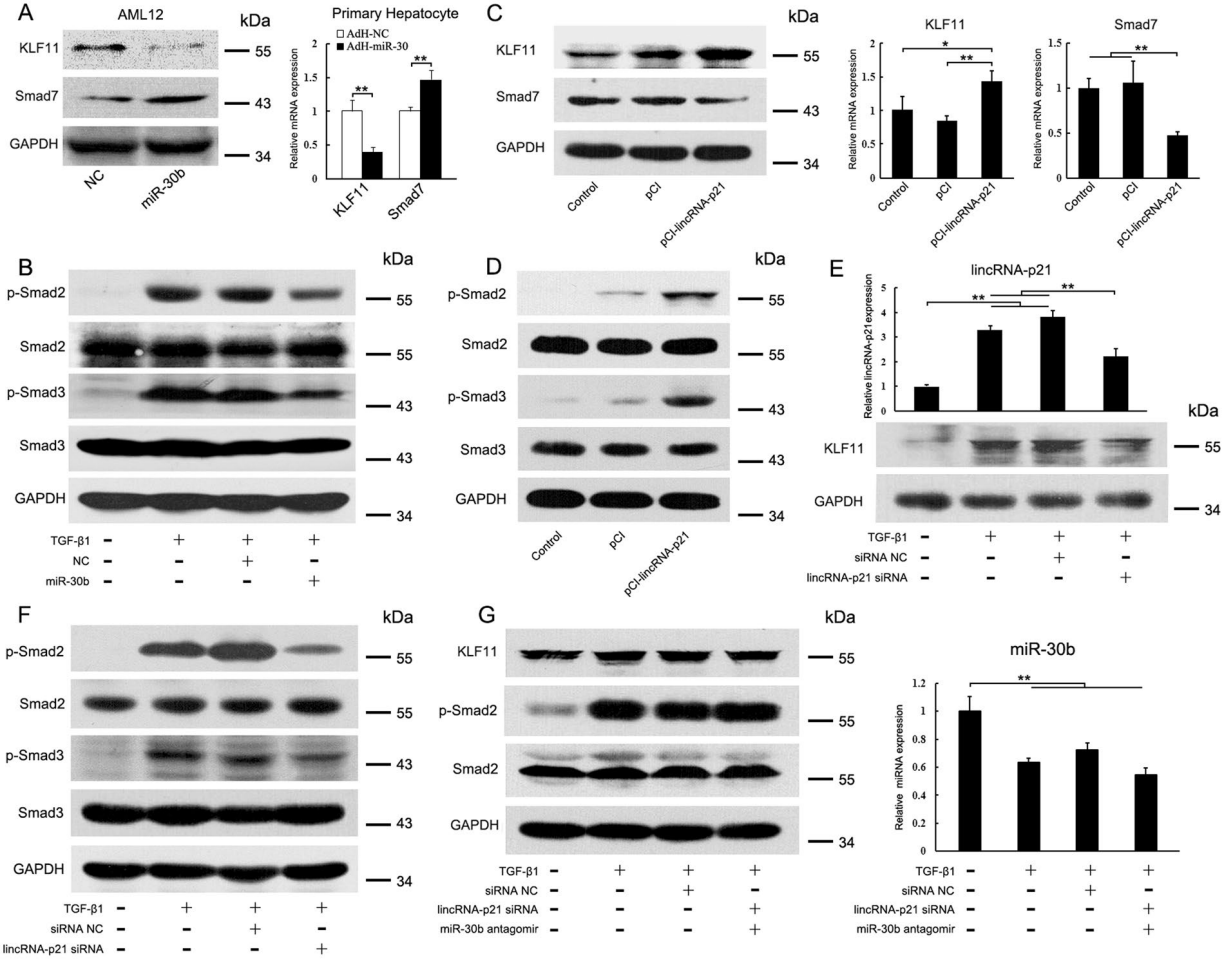
LincRNA-p21 enhances TGF-β/Smad signaling in hepatocyte by interacting with miR-30. (**A**) Effects of miR-30 on KLF11 and Smad7 expressions were determined by western blot and qRT-PCR. Left, AML12 were transfected with miR-30b mimics for 24 h. Right, primary hepatocytes were isolated from fibrotic liver injected with AdH-NC or AdH-miR-30. (**B**) miR-30 inhibits TGF-β/Smad signaling in hepatocyte. AML12 cells were transfected with miR-30b mimics, changed to serum-free DMEM for 24 h and then treated with TGF-β1 for 2 h. p-Smad2, p-Smad3 and total Smad2, Smad3 were detected by western blot. (**C**) Ectopic expression of lincRNA-p21 increases KLF11 expression in hepatocyte. AML12 cells were transfected with pCI-lincRNA-p21 for 24 h. KLF11 and Smad7 expressions were determined by western blot and qRT-PCR. (**D**) Ectopic expression of lincRNA-p21 enhances TGF-β/Smad signaling in hepatocyte. AML12 cells were transfected with pCI-lincRNA-p21 for 24 h and then treated with TGF-β1 for 2 h. (**E**) Knockdown of lincRNA-p21 reduces KLF11 expression in hepatocyte. AML12 cells were transfected with lincRNA-p21 siRNA for 24 h in the presence of TGF-β1. Top, lincRNA-p21 was determined by qRT-PCR. Bottom, KLF11 was detected by western blot. (**F**) Knockdown of lincRNA-p21 inhibits TGF-β/Smad signaling. AML12 cells were transfected with lincRNA-p21 siRNA for 24 h and then treated with TGF-β1 for 2 h. (**G**) Inhibition of miR-30 impairs the effects of lincRNA-p21 siRNA on TGF-β/Smad signaling. AML12 cells were co-transfected with lincRNA-p21 siRNA and miR-30b antagomir for 24 h and then treated with TGF-β1 for 2 h. Left, p-Smad2 and total Smad2 were detected by western blot; Right, miR-30b levels were determined by qRT-PCR. The results are shown as fold change compared with control group. Data are the mean ± SEM of three independent experiments. *P < 0.05, **P < 0.01. The original immunoblot images are shown in Supplementary Figure S10.

In our previous study, we found that miR-30 blunted TGF-β/Smad signaling in HSCs by targeting KLF11, which suppressed the transcription of inhibitory Smad7 in TGF-β/Smad pathway^31^. Here, we further revealed that the inhibition of KLF11 by miR-30 resulted in the upregulation of Smad7 in hepatocytes (Fig. 7A). In the isolated hepatocytes from fibrotic liver injected with AdH-miR-30, ectopic expression of miR-30b led to decrease of KLF11 and increase of Smad7 in hepatocyte *in vivo* (Fig. 7A). Western blot showed that miR-30b greatly inhibited the phosphorylation of Smad2 and Smad3 in TGF-β1-treated AML12 cells (Fig. 7B). Collectively, these results provide convincing evidence that miR-30 can suppress TGF-β/Smad signaling by targeting KLF11 in hepatocyte.

We next sought to investigate whether lincRNA-p21 is involved in TGFβ/Smad signaling through regulating KLF11. Luciferase reporter containing the wild or mutant KLF11 3′UTR was transfected into AML12 cells together with pCI-lincRNA-p21. Ectopic expression of lincRNA-p21 induced the increase of luciferase activity in the reporter containing the KLF11 3′UTR (Supplementary Figure S5A). However, the increase of luciferase activity was suppressed by miR-30b (Supplementary Figure S5B). Reciprocally, knockdown of lincRNA-p21 reduced the luciferase activity. The suppression of luciferase activity by lincRNA-p21 siRNA was reversed by miR-30 antagomir (Supplementary Figure S5C).

Finally, we transfected AML12 cells with pCI-lincRNA-p21, showing that ectopic expression of lincRNA-p21 increased KLF11 production, led to the suppression of Smad7 (Fig. 7C), and therefore enhanced TGFβ-induced phosphorylation of Smad2 and Smad3 (Fig. 7D). Reciprocally, delivery of lincRNA-p21 siRNA significantly suppressed the upregulation of lincRNA-p21 and led to a decrease of KLF11 in the TGFβ-treated AML12 cells (Fig. 7E). As a consequence, inhibition of lincRNA-p21 suppressed TGFβ-induced phosphorylation of Smad2 and Smad3 (Fig. 7F). To further validate that lincRNA-p21 regulates TGFβ/Smad signaling through interacting with miR-30, we co-transfected lincRNA-p21 siRNA with miR-30 antagomir, showing that lincRNA-p21 siRNA failed to reduce KLF11 expression and suppress TGFβ/Smad signaling when miR-30 was inhibited (Fig. 7G).

### LincRNA-p21 promotes hepatocyte apoptosis

**Figure 8:**
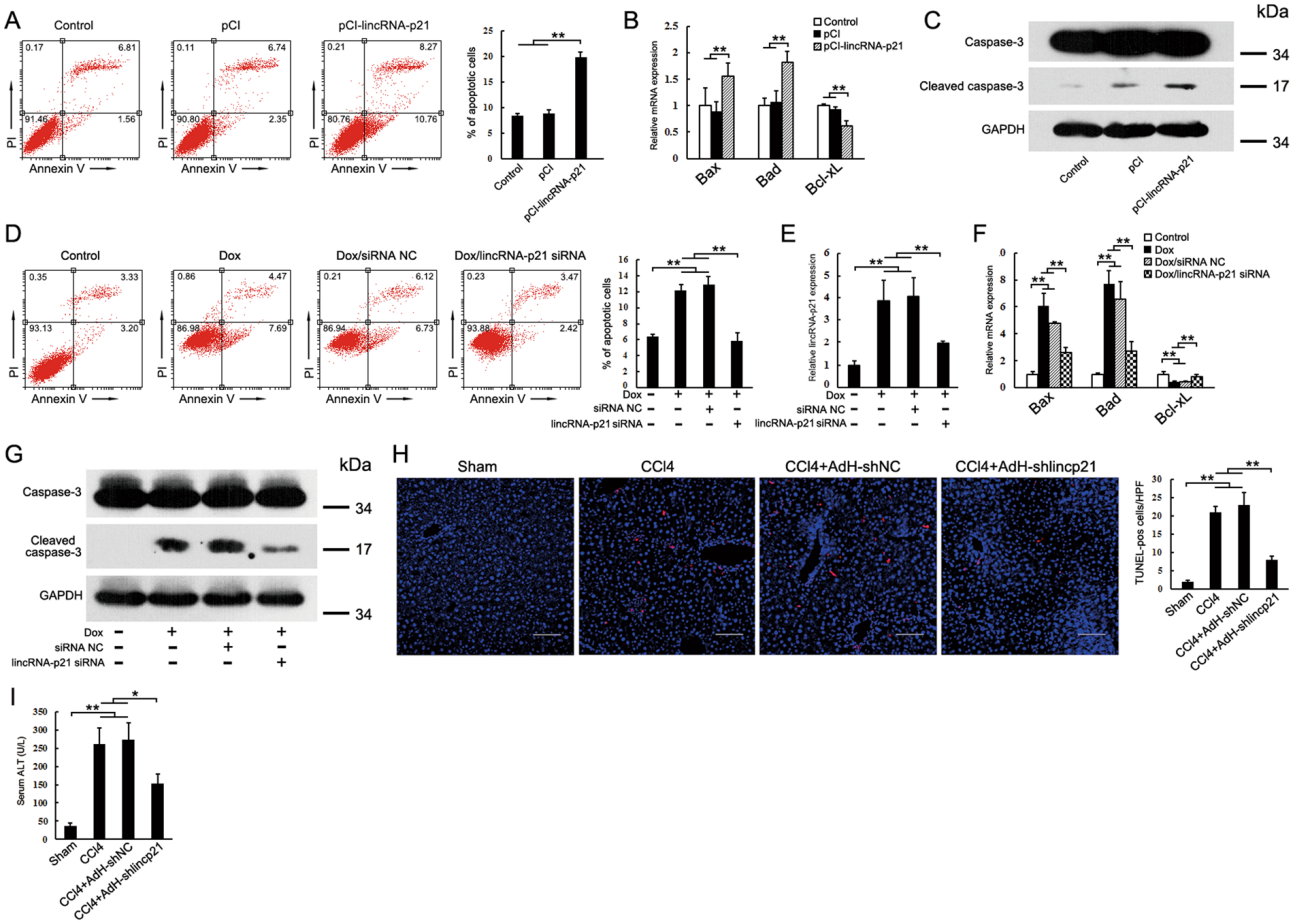
LincRNA-p21 promotes hepatocyte apoptosis. (**A**) Ectopic expression of lincRNA-p21 promotes hepatocyte apoptosis. AML12 cells were transfected with pCI-lincRNA-p21 for 48 h. Cell apoptosis was determined by FACS analysis. (**B**) Effects of lincRNA-p21 on Bax, Bad and Bcl-XL expression in AML12 cells. (**C**) lincRNA-p21 promotes caspase-3 cleavage. Caspase-3 and cleaved caspase-3 were detected by western blot. (**D**) Knockdown of lincRNA-p21 inhibits doxorubicin-induced hepatocyte apoptosis. AML12 cells were transfected with lincRNA-p21 siRNA for 48 h in the presence of 0.8 μg/ml doxorubicin. (**E**) lincRNA-p21 siRNA inhibits doxorubicin-induced upregulation of lincRNA-p21. (**F**) Effects of lincRNA-p21 siRNA on doxorubicin-induced deregulation of Bax, Bad and Bcl-XL. (**G**) Knockdown of lincRNA-p21 inhibits caspase-3 activation. (**H**) Knockdown of lincRNA-p21 inhibits hepatocyte apoptosis in the CCl_4_-induced fibrotic livers. Apoptotic hepatocytes were evaluated by TUNEL staining (left) and its quantification (right). Scale bar, 50 μm. (**I**) Knockdown of lincRNA-p21 decreased serum ALT in the CCl_4_-treated mice. qRT-PCR results are shown as fold change compared with control group. Data are the mean ± SEM of three independent experiments. *P < 0.05, **P < 0.01. The original immunoblot images are shown in Supplementary Figure S11.

The role of lincRNA-p21 in apoptosis remains controversial^19, 20, 36^. Here, we found that ectopic expression of lincRNA-p21 led to upregulation of proapoptotic genes (Bax and Bad) and the decrease of antiapoptotic Bcl-XL, and enhanced hepatocyte apoptosis (Fig. 8A and B). Western blot demonstrated that lincRNA-p21 increased caspase 3 cleavage (Fig. 8C). Since lincRNA-p21 was first identified in response to DNA damage treatment with doxorubicin (Dox), we examined the effect of lincRNA-p21 depletion on Dox-induced hepatocyte apoptosis, showing that lincRNA-p21 siRNA significantly decreased the number of Dox-induced apoptotic cells (Fig. 8D). LincRNA-p21 siRNA inhibited the increase of Dox-induced lincRNA-p21 (Fig. 8E), and led to the decrease of proapoptotic genes and the increase of antiapoptotic Bcl-XL (Fig. 8F). Additionally, lincRNA-p21 siRNA blunted the cleavage of caspase 3 (Fig. 8G).

Given that lincRNA-p21 is highly inducible by TGFβ, we explored the involvement of lincRNA-p21 in TGFβ-induced hepatocyte apoptosis. Knockdown of lincRNA-p21 significantly inhibited the TGFβ-induced apoptosis and led to the decrease of proapoptotic genes and the increase of anti-apoptotic Bcl-XL (Supplementary Figure S6A and B). In the TGFβ-induced apoptotic AML12 cells, the cleavage of caspase 3 was inhibited by lincRNA-p21 siRNA (Supplementary Figure S6C).

Finally, we investigated the *in vivo* role of lincRNA-p21 in hepatocyte apoptosis. Hepatocyte apoptosis remarkably increased in CCl_4_-induced fibrotic liver as assessed by counting the number of apoptotic terminal deoxynucleotidyl transferase–mediated deoxyuridine triphosphate nick-end labeling (TUNEL)-positive hepatocytes. However, knockdown of lincRNA-p21 blunted the increase of hepatocyte apoptosis (Fig. 8H). We also determined the serum alanine aminotransferase (ALT) levels, showing that knockdown of lincRNA-p21 significantly decreased serum ALT in the CCl_4_-treated mice (Fig. 8I).

### LincRNA-p21 contributes to regulating hepatocyte growth in fibrotic liver

Increased hepatocyte death and growth inhibition enhance the secretion of pro-inflammatory and profibrogenic cytokines. We found that enforced expression of lincRNA-p21 in AML12 cells resulted in a significant decrease in viability (Supplementary Figure S7A). Conversely, knockdown of lincRNA-p21 increased AML12 cell viability (Supplementary Figure S7B). In TGFβ-treated AML12 cells, lincRNA-p21 siRNA prevented the TGF-β-induced hepatocyte death (Supplementary Figure S7C). A similar observation was made in cells treated with Dox (Supplementary Figure S7D). Additionally, proliferating hepatocytes significantly increased in livers from AdH-shlincp21 group compared with those of AdH-shNC group as evaluated by immunohistochemical staining of Ki67 (Supplementary Figure S7E).

## Discussion

lncRNAs play crucial roles in specific cell types, tissues and developmental conditions by various mechanisms. Correspondingly, most lncRNAs exhibit tissue or cell-specific expression^37^. Here, using RNA sequence, we identified differentially expressed lincRNA-p21 in hepatocyte during liver fibrosis. Similarly, lincRNA-p21 increased in the livers of mice treated with the carcinogen furan, and thus implying an extensive role of lincRNA-p21 in the response to liver injury^38^. Of note, lincRNA-p21 also increased and contributed to pulmonary fibrosis in acute respiratory distress syndrome^39^. However, the deregulation of lincRNA-p21 in the progression of fibrosis still remains elusive. A recent report showed that lincRNA-p21 decreased in human cirrhotic liver and a higher dose CCl_4_-induced fibrotic liver^40^. Given the fact that lncRNAs are poorly conserved among species, more investigations are needed to confirm the regulation of lincRNA-p21 under different pathological conditions.

Previous reports showed that the transcription of lincRNA-p21 is regulated by various signaling pathways under different conditions^18, 21^. Here, using both *in vitro* and *in vivo* studies, we observed a significant increase of hepatocyte lincRNA-p21 after TGF-β1 treatment, and identified the promoter region responsible for TGF-β/Smad signaling. Since TGF-β1 increased during liver fibrogenesis triggered by various causes, it’s tempting to speculate that TGF-β1-mediated upregulation of lincRNA-p21 may work as a specific downstream effector of TGF-β signaling in hepatocyte, and therefore represent a hitherto unknown paradigm in liver fibrosis.

miRNAs with profibrogenic or antifibrogenic functions have been implicated in diverse animal models and human patients. However, few studies identified their roles in hepatocyte. Here, our results demonstrate that hepatocyte miR-30 greatly inhibits fibrotic TGF-β/Smad signaling by targeting KLF11 and consequently prevents liver fibrosis. KLF11 isn’t a member of Smad family. However, the induction of KLF11 strengthens TGF-β/Smad signaling cascade through suppressing the Smad7 expression. In addition, previous studies showed that KLF11 overexpression mimics the TGF-β-induced effects in different types of cells^41, 42^. Taken together, inhibition of KLF11 could be an effective way to suppress the excessive TGF-β/Smad signaling during liver fobrosis.

The increase of lincRNA-p21 in hepatocyte was associated with the loss of miR-30 during liver fibrosis. In both *in vitro* and *in vivo* systems, we observed that downregulation of lincRNA-p21 was sufficient to suppressed TGF-β signaling and liver fibrosis. These phenomena depend on the interaction between lincRNA-p21 and miR-30. The presence of competitive miR-30 antagomir abolished the inhibitory effects of lincRNA-p21 knockdown on TGF-β signaling and liver fibrogenesis, indicating that lincRNA-p21 functions as a ceRNA. Basing on these results, we propose that TGF-β-induced lincRNA-p21 in turn strengthens TGF-β signaling by interacting with miR-30, thus forming a positive feedback loop to ensure lincRNA-p21 expression and mediate the role of TGF-β in promoting liver fibrosis.

To date, the mechanism of miR-30 deregulation in various states is mostly unknown. Here, we provide the first evidence that TGF-β-induced lincRNA-p21 inhibited miR-30 by directly binding to them. Moreover, the transcribing of pri-miR-30 wasn’t affected by TGF-β, and thus strongly suggesting the underlying mechanism responsible for miR-30 decrease in response to TGF-β. However, at this stage, we can’t exclude the possibility that the decrease of miR-30 may be triggered by other mechanisms in liver fibrosis. To clarify this issue, further studies will be needed.

Hepatocyte apoptosis and cell death is a key trigger of liver fibrogenesis^7, 8^. To date, a number of lncRNAs have been shown to modulate apoptosis^43, 44^. However, there are conflicting reports on the role of lincRNA-p21 in apoptosis and cell growth^19, 20, 36^. Here, *in vitro* and *in vivo* results confirmed the contribution of lincRNA-p21 to hepatocyte apoptosis during liver fibrosis, showing that inhibition of hepatocyte lincRNA-p21 reduced hepatocyte death and prevented subsequent inflammatory cell recruitment and activation of inflammatory and fibrogenic signal cascade. LincRNA-p21 was initially identified as a transcriptional target of p53. Subsequent investigations suggested that lincRNA-p21 functioned as a downstream repressor in the p53 transcriptional response and promoted p53-mediated expression of p21^20^. Notably, hepatocyte-specific deletion of p53 decreased liver fibrosis through downregulating the profibrogenic mediators^45^. Thus, whether hepatocyte lincRNA-p21 functions as a more general mediator of multiple profibrogenic signaling pathways remains to be defined.

In summary, our present results extend the knowledge of lincRNA-p21 and deepen the understanding of the cross talk between lncRNA and profibrogenic TGF-β signaling cascades in hepatocyte during liver fibrosis. The hypothetical roles of hepatocyte lincRNA-p21 in TGF-β signaling and liver fibrosis are schematically summarized in Supplementary Figure 8. The pleiotropic effects of hepatocyte lincRNA-p21 on liver fibrosis suggest that it could be an effective target for therapy.

## Materials and Methods

### Antibodies and Reagents

The antibodies used in the study are listed in Supplementary Table 1. Recombinant human TGF-β1 was from Peprotech (Rocky Hill, NJ). Other reagents were purchased from Sigma-Aldrich (St. Louis, MO) unless otherwise indicated.

### Cell culture

Cells were incubated in a humidified atmosphere of 5% CO_2_ at 37 °C_._ In this study, recombinant TGF-β1 was added to the medium at a final concentration of 5 ng/ml or as indicated. The culture medium and isolation of hepatocyte and HSC were described in the Supplementary Materials and Methods.

### Flow Cytometry Analysis

AML12 cells from different groups were collected and stained with FITC-labeled Annexin V and PI according to the manufacturer’s instructions (eBioscience, San Diego, CA). The percentage of apoptotic cells was determined by a FACScan flow cytometer (BD Biosciences, San Jose, CA).

### Animal treatments

Animal protocols were reviewed and approved by the Animal Care and Use Committee of Nanjing University, and conformed to the Guidelines for the Care and Use of Laboratory Animals published by the National Institutes of Health. Five-week old male C57 mice (20 ± 2 g) were obtained from the Animal Center of Yangzhou University (Yangzhou, China). Animals were maintained under pathogen-limited conditions and had free access to rodent chow and water. Mouse liver fibrosis was induced according to previously described mothed^31^. Briefly, mice were intraperitoneally injected with of 20% CCl_4_ solution in sterile mineral oil at a dose of 2.5 ml CCl_4_ per kilogram body weight twice per week for three or four weeks. The adenovirus was injected only one time via tail vein at two days before the first CCl_4_ injection (1 × 10^9^ pfu/mouse).

### Histological examination

Histological examinations of liver were performed as described before^31^ in Supplementary Materials and Methods.

### RNA isolation and quantitative real-time PCR

Total RNA was extracted from the cells or liver tissues using TRIzol Reagent (Invitrogen) and checked for purity and concentration with A_260_/A_280_ reading (Biophotometer Plus, Eppendorf, Germany). Quantitative real-time PCR was performed as described in Supplementary Materials and Methods. Primers are listed in Supplementary Table 2.

### Northern blot

LincRNA-p21 probe was labelled with digoxigenin (DIG) using a DIG DNA Labelling Kit (Roche). Total RNA extracted from AML12 cells and run on a 1% denatured agarose gel, transferred to positively charged nylon membranes (Millipore) followed by cross-linking through UV irradiation. The membrane was then hybridized with (DIG)-labelled probe overnight. The detection was performed using a DIG luminescent detection kit (Roche) according to the manufacturer’s instructions.

### Western blot

Western blot was performed as described before^31^. In brief, the cell lysates were separated by SDS-PAGE and then transferred onto PVDF membranes. After incubation with primary antibody, the membranes were washed with PBST and then probed with horseradish peroxidase-conjugated secondary antibody at room temperature. The immunoreactive bands were detected by fluorography using an enhanced chemiluminescence system (Cell Signaling Technology, Beverly, MA).

### Statistical Analysis

Results in this study are expressed as the means ± standard error of the mean (SEM). The data were analyzed for normal distribution. Differences between multiple groups were checked using one-way ANOVA with post-hoc Bonferroni correction. Differences between two groups were analyzed by a two-tailed unpaired Student’s t test. A value of P < 0.05 was considered statistically significant, and P < 0.01 indicated strongly significant difference.

Other methods used in this study are described in the Supplementary Materials and Methods.

## Electronic supplementary material

Supplementary file
