# Supplementary material for: TGF-β-induced hepatocyte lincRNA-p21 contributes to liver fibrosis in mice
Source: Sci Rep. 2017 Jun 7;7:2957. doi: 10.1038/s41598-017-03175-0 (PMC5462818; doi:10.1038/s41598-017-03175-0)

TGF-β-induced hepatocyte lincRNA-p21 contributes to liver fibrosis in mice

Xiaolong Tu[1, #], Yuanyuan Zhang[1, #], Xiuxiu Zheng[1, #], Jia Deng[1], Huanan Li[1], Zhiqian Kang[1], Zhipeng Cao[1], Zhen Huang[1], Zhi Ding[1], Lei Dong[1], Jiangning Chen[1], Yuhui Zang[1, *], Junfeng Zhang[1, 2, *]

1 *State Key Laboratory of Pharmaceutical Biotechnology, School of Life Science, Nanjing University, Nanjing 210093, PR China*
2 *Jiangsu Engineering Research Center for microRNA Biology and Biotechnology, Nanjing 210093, PR China*

# These authors contributed equally to this work.

*** Corresponding authors:** Yuhui Zang, State Key Laboratory of Pharmaceutical Biotechnology, School of Life Sciences, Nanjing University, Nanjing 210093, PR China. Email: zangyh@nju.edu.cn, Tel: 86-25-89681321, Fax: 86-25-83324605; or Junfeng Zhang, State Key Laboratory of Pharmaceutical Biotechnology, School of Life Sciences, Nanjing University, Nanjing 210093, PR China. Email: jfzhang@nju.edu.cn, Tel: 86-25-89681317, Fax: 86-25-83596239.

Supplementary Materials and Methods

Oligonucleotides and Transfection

miRNA duplexes corresponding to mature miR-30, the antagomirs and their negative control (NC) were purchased from Genepharma Biotechnology (Shanghai, China). The negative control RNA for miRNA mimics or antagomirs was nonhomologous to any mouse genome sequence. lincRNA-p21-specific siRNA oligonucleotides (Sense: 5’- AAAUAAAGAUGGUGGAAUGTT-3’ and antisense: 5’- CAUUCCACCAUCUUUAUUUTT-3’) were obtained from GenePharma. The phosphorothioate-modified antisense oligonucleotides specific for lincRNA-p21 (5’-GCCATCCTCGGTACTGAGGT-3’) and the scrambled control (5’-CCTTCCCTGAAGGTTCCTCC’) were synthesized by SBS Gene Biotechnology (Shanghai, China). The phosphorothioate-modified antisense oligonucleotides specificformiR-30 (5’-GCTGAGTGTAGGATGTTTAC-3’) and the scrambled control (5’-ACGTGACACGTTCGGAGAA-3’) were also synthesized by SBS Gene Biotechnology. Transfection of RNA duplex was performed using Lipofectamine 2000 (Invitrogen). Unless otherwise indicated, the cells were transfected with the transfection mix containing 50 nM of miRNA duplex, 100 nM of miRNA inhibitor or 100 nM of siRNA.

Cell culture

Primary hepatocytes were isolated by pronase/collagenase perfusion digestion followed by subsequent density gradient centrifugation as previously described [1]. The nontumorigenic mouse hepatocyte cell line AML-12 was maintained in DMEM/F12 medium (Invitrogen, Camarillo, CA) containing 10% fetal calf serum, 1×ITS (insulin-transferrin-sodium selenite media supplement,

Sigma-Aldrich), dexamethasone (40 ng/ml), penicillin (100 units/ml), and streptomycin (100 μg/ml). The rat hepatic stellate cell line HSC-T6 and the human embryonic kidney cell line 293A were cultured in high-glucose DMEM supplemented with 10% fetal calf serum. All cells were incubated at 37 ℃ in a humidified atmosphere of 5% CO_2. In the case of TGF-β1 treatment, recombinant human TGF-β1 was added at a final concentration of 5 ng/ml, or as indicated. TGF-β receptor inhibitor SB431542 was added at a final concentration of 2 μM.

Luciferase reporter assay

To perform luciferase reporter assay, HEK293T or AML12 cells grown in a 24-well plate were co-transfected with 200 ng of recombinant pMIR-REPORT, 2 ng of pMIR-REPORT β-gal control Plasmid that is designed for transfection normalization and miRNA mimics, incubated for 24 h. After incubation, the Dual-Glo Luciferase Assay System (Promega, Beijing, China) was used to analyze luciferase expression according to the manufacturer's protocol. Firefly luciferase activity was normalized to β-galactosidase expression to adjust for variations in transfection efficiency among experiments.

Cell proliferation assay

AML12 cells were seeded on 96-well plate in triplicate at an initial number of 2,000 cells per well, grown to approximately 70% confluence and transfected with plasmid or siRNA. After the transfected cells were incubated for the indicated times, cell proliferation was determined using the Cell Counting kit-8 (Byeotime, Nantong, China) according to the manufacturer's protocol. The A450 absorbance values were determined on a double-channel Microplate Reader (Tecan Group

Ltd., Männedorf, Switzerland). Cell proliferation was calculated on the basis of absorbency.

Plasmids

The cDNA encoding lincRNA-p21 was amplified by RT-PCR with primers (Forward: 5’-TGGCAGTCTGACCCACACTCC-3’ and Reverse: 5’-ACACGTGTGTATGATTGTCTGTGC-3’). The PCR product was cloned into pEasy-T1 (TransGen Biotech, Beijing, China), downstream of T7 promoter to yield pEasy-T1-lincRNA-p21. pEasy-T1-lincRNA-p21Mut, which carried mutated putative binding sites for miR-30, was generated by site-specific mutagenesis using MutanBEST Kit (Takara, Dalian, China) in according to the manufacturer’s instruction. The cDNA coding antisense lincRNA-p21 transcripts (negative control) was also cloned downstream of T7 promoter to yield pEasy-T1-lincRNA-p21R. The validated lincRNA-p21 cDNA was then cloned into the mammalian expression vector pCI (Promega, Madison, WI) to yield pCI-lincRNA-p21. The cDNA fragments of lincRNA-p21 that contained putative binding sites for miR-30 was obtained by PCR primers (Forward: 5’-GCGAGCTCCGTCTCCAGTTCCTA-3’ and Reverse: 5’-GCACGCGTGAGCATGAGACTCCTG-3’). The amplified fragments was inserted in to pMD18-T vector (Takara), sequenced and then cloned downstream of the firefly luciferase coding region in the pMIR-REPORT (Ambion, Grand Island, NY). The mutant 3’ UTR fragments which carried mutated putative binding sites for miR-30 was generated by site-specific mutagenesis using MutanBEST Kit (Takara) in the pMD18-T vector.

A set of selected fragments containing putative lincRNA-p21 promoter region were amplified by PCR with primers were as follows: -1825 Forward: 5’-CCTCAGCTACTCCTTTGTCCTGTAA-3’;

-1426 Forward: 5’-CGTTCTGATGCTCTTCTGACATGC-3’; -780 Forward: 5’-GTCGACATACACTGGAACAAGACA-3’; and +115 Reverse: 5’-CAAAGCTGGGAGATGATGCAACCGA-3’. The PCR fragments were inserted into pMD18-T vector, sequenced and then cloned into luciferase reporter vector pGL3-basic (Promega, Madison, WI) between KpnI-HindIII sites. We also amplified the fragment with the predicted Smad binding sites (-1825 to -1426) with primer -1825 Forward: 5’-CCTCAGCTACTCCTTTGTCCTGTAA-3’ and -1426 Reverse: 5’-CATTATAAATGGTTGACAGCCACTA-3’, and inserted the fragment into pMD18-T vector. The mutant -1825 to -1426 fragment, in which the nucleotide sequence of Smad binding site was mutated from AGACAGCTC to ActCtGtTa, was generated using MutanBEST Kit (Takara). The wild and mutant -1825 to -1426 fragments were then cloned into luciferase reporter vector pGL3-promoter (Promega) between KpnI-NheI sites respectively.

Generation of virus

The 2.4 kb mouse albumin promoter contains an upstream enhancer region (-10400 to-8500) and the 335-base-pair core region of ALB promoter [2]. They are amplified by PCR using mouse genomic DNA as a template (Primers for enhancer, Forward: 5’-GCTAGCTTCCTTAGCATGACGTT -3’ and Reverse: 5’-GGATCCCAAGCTGGAGAACGAGT -3’; Primers for core region of promoter, Forward: 5’-ATGTGGCATGCTTCCATGCCAAG -3’ and Reverse: 5’-GCTAGTGGGGTTGATAGGAAAGGT-3’). The amplified fragments were fused together and inserted in to pMD18-T vector. The recombinant adenovirus for ectopic expression of miR-30b (AdH-miR-30) and scrambled controls (AdH-NC) were generated using BLOCK-iT Adenoviral

RNAi Expression System (Invitrogen, Life Technologies), in which the U6 promoter of the vector was replaced by the 2.4 kb mouse albumin promoter fragment. The adenovirus for specific lincRNA-p21shRNA expression in hepatocytes was generated in a similar way. The target sequence is AAAUAAAGAUGGUGGAAUG. All adenoviruses were prepared by Life Technologies (Shanghai, China). After large-scale preparation, adenoviruses were concentrated by ViraBind Adenovirus Purification Kit (Cell Biolabs, San Diego, USA), dialyzed in phosphate buffered saline (PBS) and stored in aliquots at -80 ℃ until use. The titer of each virus stock was determined by plaque assay on 293 cells.

Measurement of hepatic hydroxyproline content

The amount of hydroxyproline was determined with Hydroxyproline Testing Kit (Jiancheng, Nanjing, China). About 100 mg wet liver samples were weighed and subjected to acid hydrolysis. The hepatic hydroxyproline levels were then biochemically measured and calculated according to the manufacturer's instruction.

RNA isolation and quantitative real-time PCR

Total RNA was prepared from the cells or liver tissues by homogenization and purification using TRIzol Reagent (Invitrogen, Carlsbad, CA, USA). For mRNA detection, 500 ng of total RNA were used for complementary DNA synthesis and realtime PCR was performed in triplicates using the SYBR PrimeScript RT-PCR Kit (Takara) in an ABI 7300 Fast Realtime PCR System (Applied Biosystems, Foster City, CA) according to the manufacturer's instructions. Individual gene expression was quantified using a standard curve and normalized to β-actin mRNA expression.

The PCR conditions were as follows: 95 ℃ for 30 s, and then 40 cycles of amplification for 5 s at 95 ℃ and 30 s at 60 ℃ Primers are listed in Supplementary Materials. For analysis of miR-30 levels, 2 μg of total RNA were used for first-strand DNA synthesis with a microRNA specific oligonucleotides. Realtime PCR was carried out by the SYBR Green PCR method at 95 ℃ for 10 min, followed by 40 cycles of amplification at 95 ℃ for 15 s and 60 ℃ for 60 s. The U6 snRNA level was measured and used to normalize the relative abundance of miRNA. Each sample was analyzed in triplicates and repeated for three or four independent assays. Real-time qPCR was performed on a 7300 Sequence Detection System (Applied Biosystems).

Absolute quantification using real-time RT-PCR.

The absolute copy numbers of lincRNA-p21 and miR-30 were measured using the standard curve method as previously described [3]. Briefly, pEasy-T1-lincRNA-p21 was linearized and generated sense RNA transcript using in vitro T7 promoter transcription system (Promega). After digested with RNAse-free DNAse and purification, the transcript was quantified on a spectrophotometer and converted to the number of copies using the previously described method [3]. The quantified lincRNA-p21 RNA was used as the standard of lincRNA-p21. Synthetic miR-30 RNA was used as the standard of miR-30. The lincRNA-p21 and miR-30 standards RNA, as well as the total RNA of AML12 cells, were reversely transcribed respectively using the reverse primer. The standard cDNAs were serially diluted in nuclease-free water. Serial dilutions from 10^6 to 10^1 copies were used for standard in a final volume of 20 μl. Quantitative PCR was then performed using the diluted cDNA standards or total cDNA from AML12 as templates. Absolute quantification determines the actual copy numbers of target genes by relating the Ct value to a standard curve.

The final data were expressed as the copy number per 10 pg total RNA.

RNA pull-down with biotinylated lincRNA-p21

Biotin-labeled lincRNA-p21 or incRNA-p21 mutant harboring mutated miR-30 binding site were *in vitro* transcribed respectively from plasmid pEAsy-T1-lincRNA-p21 or pEAsy-T1-lincRNA-p21Mut, with the Biotin RNA Labeling Mix (Roche, Indianapolis, IN, USA) and T7 RNA polymerase (Promega), treated with RNase-free DNase I (Promega), and purified with an RNeasy Mini Kit (Qiagen, Valencia, CA). 3 μg of biotinylated RNA in RNA structure buffer (10 mM Tris pH 7, 0.1 M KCl, 10 mM MgCl2) was heated to 95 ℃ for 2 min, put on ice for 3 min, and then left at room temperature (RT) for 30 min to allow proper secondary structure formation. One milligram of whole-cell lysates from AML12 cells were incubated with folded transcripts for 1 hr at 25 ℃. Complexes were isolated with Streptavidin agarose beads (Invitrogen). The RNA present in the pull-down material was detected by qRT-PCR analysis.

RNA Pull-down assay with biotinylated miR-30.

Biotinylated miR-30b was synthesized by Genepharm. The pull-down assay was carried out as previously described [3]. Briefly, 50 nM biotinylated miR-30b was transfected into AML12 cells. The cells were then harvested 48 h after transfection and washed with PBS followed by incubation in a lysis buffer (20mMTris, pH 7.5, 200mM NaCl, 2.5mM MgCl2, 0.05% Igepal, 60Uml^{-1}Superase-In, 1mM DTT, protease inhibitors (Roche)) on ice for 10min. The lysates were precleared by centrifugation and incubated with Streptavidin agarose beads (Invitrogen) that were pre-coated with RNase-free BSA and yeast tRNA (both from Sigma). The bound RNAs were then

purified by Trizol for the analysis.

ChIP

ChIP assay was performed by Chromatin Immunoprecipitation Assay Kit (Millipore, Bedford, MA, USA) as described previously [4]. In brief, AML12 cells pretreated with TGF-β1 were firstly cross-linked with 1% formaldehyde for 10 minutes at 37℃, then quenched with glycine, and sonicated to generate 300- to 600-bp DNA fragments. Immunoprecipitation was performed with Smad3 antibody and normal isotype IgG was used as a control. Precipitated DNAs were detected by PCR using specific primers: 5’- CTCTCCCTCATATCTACA-3’ and 5’-GTATGGTGTTCTATCTGC-3’.

Histological examination

Excised liver tissues were fixed in 4% paraformaldehyde, embedded in paraffin and sectioned. The tissue was then stained with hematoxylin–eosin (H&E) or Sirius red. For semi-quantitative analysis of liver fibrosis, ten randomly selected fields per liver section of each mouse were recorded, and the Sirius red-stained areas were quantified using Image-Pro Plus6.0 (Media Cybernetics, Bethesda, MD).

Immunofluorescence Staining

For immunofluorescence staining of tissue, paraffin-embedded liver tissue were sectioned and incubated with the primary antibody at 4℃ overnight according to the manufacturer’s recommendation. After the sections were washed with Tris-buffered saline, they were subsequently stained with FITC-conjugated secondary antibodies (Santa Cruz) and imaged by

fluorescent microscopy. The nuclei were counterstained with 4,6-diamidino-2-phenylindole (DAPI). For immunofluorescence staining of cell, primary or cultured cells were seeded on 8-well Millicell EZ slide (Millipore) and treated as indicated. The slides were washed with PBS, followed by fixing in 100% methanol at room temperature for 15 minutes. After being blocked with 2.5% bovine serum albumin, the samples were probed with appropriate primary antibodies. After several washes with PBS, the samples were incubated with fluorescent-conjugated secondary antibodies. The nuclei were stained with DAPI and the fluorescence was visualized under Nikon confocal microscope (C2+, Nikon, Tokyo, Japan).

Western blot

Cell pellets were lysed in lysis buffer (20 mM Tris-HCl, pH 7.5, 150 mM NaCl, 1% Triton X-100, sodium pyrophosphate, β-glycerophosphate, EDTA, Na_3VO_4 and leupeptin) containing 1 mM PMSF. After mixing with 2×Laemmli buffer and heat denaturation at 100℃ for 5 min, equal amounts of lysate (20 μl) were separated by SDS-PAGE and then transferred onto PVDF membranes. The membranes were incubated with primary antibody at appropriate dilutions for 1 h. After washing with PBST, the membrane was then probed with horseradish peroxidase-conjugated anti-rabbit or anti-goat IgG for 1 h at room temperature. The protein bands were visualized by fluorography using an enhanced chemiluminescence system (Cell Signaling Technology, Beverly, MA).

Serum aminotransferase activity measurement

The blood samples were allowed to clot, then centrifuged at 3000×g for 10 min to collect serum for aminotransferase activity analysis. Alanine aminotransferase (ALT) was measured using

commercial kits (Jiancheng, Nanjing, China) and expressed as U/L.

Reference

1. Weiskirchen R and Gressner AM. Isolation and culture of hepatic stellate cells. Methods Mol Med. 2005; 117: 99-113.
2. Srivastava J, Siddiq A, Emdad L, Santhekadur PK, Chen D, Gredler R, Astrocyte elevated gene-1 promotes hepatocarcinogenesis: novel insights from a mouse model. Hepatology 2012; 56:1782-1791.
3. Wang K, Liu CY, Zhou LY, Wang JX, Wang M, Zhao B, et al. APF lncRNA regulates autophagy and myocardial infarction by targeting miR-188-3p. Nat Commun. 2015; 6: 6779.
4. Qin W, Chung A, Huang XR, Meng Xi, Hui D, Yu C, Sung J, Lan HY. TGF-β/Smad3 Signaling Promotes Renal Fibrosis by Inhibiting miR-29. J Am Soc Nephrol 2011; 22: 1462–1474.

Supplementary Table 1. Primary and secondary antibodies used in this study

Rat antigens	Poly/mono-clonal	Manufacturer	Dilution
α-SMA	monoclonal	BOSTER Biotech, Wuhan, China	1:400
Smad7	polyclonal	Abcam, AB Biotech Company, Cambridge, MA.	1:200
KLF11	polyclonal	Santa Cruz Biotechnology, Inc., Santa Cruz, CA	1:200
Collagen I	polyclonal	Abcam, AB Biotech Company, Cambridge, MA.	1:200
F4/80	polyclonal	Abcam, AB Biotech Company, Cambridge, MA.	1:500
Smad2	polyclonal	Cell Signaling Technology Inc., Beverly, MA	1:1000
Smad3	polyclonal	Cell Signaling Technology Inc., Beverly, MA	1:1000
p-Smad2	monoclonal	Cell Signaling Technology Inc., Beverly, MA	1:1000
p-Smad3	polyclonal	Cell Signaling Technology Inc., Beverly, MA	1:1000
Caspase-3	monoclonal	BOSTER Biotech, Wuhan, China	1:200
GAPDH	monoclonal	Kangchen,Shanghai,China	1:10000
Anti-rabbit IgG, HRP-linked antibody		Cell Signaling Technology Inc., Beverly, MA	1:1000
Donkey anti-goat IgG-HRP		Santa Cruz Biotechnology, Inc., Santa Cruz, CA	1:5000
Donkey anti-goat IgG-FITC		Santa Cruz Biotechnology, Inc., Santa Cruz, CA	1:100

Supplementary Table 2 qRT-PCR primers used in this study

Smad7	Forward (5′- 3′)	ACTCCAGATACCCGATGGATTT
	Reverse (5′- 3′)	CCTCCCAGTATGCCACCAC
KLF11	Forward (5′- 3′)	ATCGAAGCTGTGGAAGCTCT
	Reverse (5′- 3′)	GCTCTGAGGAGGAGTTATACA
Col1a1	Forward (5′- 3′)	TCCGGCTCCTGCTCCTCTTAG
	Reverse (5′- 3′)	AGGCCATTGTGTATGCAGCTGAC
TGF-β1	Forward (5′- 3′)	ATACGCCTGAGTGGCTGTCT
	Reverse (5′- 3′)	TCATGGATGGTGCCCAGGTC
Bax	Forward (5′- 3′)	TTGCTGATGGCAACTTCAAC
	Reverse (5′- 3′)	GATCAGCTCGGGCACTTTAG
Bcl-XL	Forward (5′- 3′)	GTTGGATGGCCACCTATCTG
	Reverse (5′- 3′)	GTTGGATGGCCACCTATCTG
BAD	Forward (5′- 3′)	AGAGTATGTTCCAGATCCCAG
	Reverse (5′- 3′)	GTCCTCGAAAAGGGCTAAGC
TIMP-1	Forward (5′- 3′)	TTCTGCAACTCGGACCTGGT
	Reverse (5′- 3′)	GCATCTTAGTCATCTTGATCT
IL-1β	Forward (5′- 3′)	GACTGTTTCTAATGCCTTCCC
	Reverse (5′- 3′)	CATGGTTTCTTGTGACCCTGA
IL-6	Forward (5′- 3′)	TGATGCACTTGCAGAAAACA
	Reverse (5′- 3′)	ACCAGAGGAAATTTTCAATAGGC
CCL2	Forward (5′- 3′)	CCTGCTGTTCACAGTTGCC
	Reverse (5′- 3′)	ATTGGGATCATCTTGCTGGT
Albumin	Forward (5′- 3′)	TGTCAACCCCAACTCTCGTG
	Reverse (5′- 3′)	AACATGCTCACTCACTGGGG
α-SMA	Forward (5′- 3′)	TTGCTGACAGGATGCAGAAGG
	Reverse (5′- 3′)	CTGATCCACATCTGCTGGAAG
CTGF	Forward (5′- 3′)	TTTCTGGCTGCACCAGTGTGAA
	Reverse (5′- 3′)	GGACAGTTGTAATGGCAGGCAC
lincRNA-p21	Forward (5′- 3′)	CAGGGCAAGAACTTGTGGAC
	Reverse (5′- 3′)	GGAACTGGAGACGGAATGTC
β-actin	Forward (5′- 3′)	GACCTCTATGCCAACACAGTGC
	Reverse (5′- 3′)	GTACTCCTGCTTGCTGATCCAC

Supplementary Figure Legends

Supplementary Figure S1. (A) Schematic outlining the predicted binding of miR-30s with lincRNA-p21. Left, the prediction of miR-30s binding sites on lincRNA-p21. The red nucleotides are the seed sequences of miR-30s. Right, free energy of hybridization between lincRNA-p21 and miR-30s. (B) pCI-lincRNA-p21 mediates enforced expression of lincRNA-p21. AML12 cells were transfected with pCI or pCI-lincRNA-p21 for 24 h respectively. LincRNA-p21 was determined by qRT-PCR. The relative gene expression was normalized against β-actin unless noted otherwise in this study. The results are shown as fold change compared with control cells. (C) miR-30s don't exhibit significant effect on lincRNA-p21 in hepatocyte. AML12 cells were transfected with miR-30a, -30b,-30c, -30d or -30e for 48 h respectively. The results are shown as fold change compared with control cells. (D) miR-122, -18, -101a and -101b exhibited no enrichment in the lincRNA-p21 RIP. AML12 cells were harvested and mixed with biotinylated lincRNA-p21 (Biotin-lincRNA-p21) or biotinylated lincRNA-p21 Mutant (Biotin-lincRNA-p21Mut) to perform biotin-based pull down. miR-122a, -101a, -21, and -18a enrichments were determined by qRT-PCR and normalized to control. Data are the mean ±SEM of three independent experiments. NS, no significant change.

Supplementary Figure S2. (A) Regulation of miR-30 in hepatocyte during CCl_4-induced liver fibrosis. miR-30a, -30b, -30c, -30d and -30e in the hepatocytes isolated from CCl_4-induced fibrotic liver were determined every week by qRT-PCR. The miRNA levels were normalized against U6 snRNA unless noted otherwise in this study. The results are shown as fold change compared with oil-treated mouse. (B) AdH-miR-30 specifically mediates the overexpression of

miR-30b in hepatocyte. miR-30b levels were determined by qPCR in mouse hepatocytes cell line AML12 and rat HSC cell line HSC-T6 that were infected with AdH-NC or AdH-miR-30 for 48 h. The results are shown as fold change compared with cells infected with AdH-NC. Data are the mean ±SEM of three independent experiments. ***P* < 0.01. NS, no significant change.

Supplementary Figure S3. (A) AdH-shlincp21 specifically decreases lincRNA-p21 in hepatocyte. LincRNA-p21 transcripts were determined by qRT-PCR in mouse hepatocytes cell line AML12 and rat HSC cell line HSC-T6 that were infected with AdH-shNC or AdH-shlincp21 for 48 h. (B) Representative images of immunofluorescence staining with anti-F4/80 antibody. Liver sections from CCl4+AdH-shNC and CCl4+AdH-shlincp21 groups were stained with antibodies specific for F4/80 (green). Nuclei were visualized using DAPI staining (blue). Scale bar, 20 μm. Data are the mean ±SEM of three independent experiments. ***P* < 0.01. NS, no significant change.

Supplementary Figure S4. (A) SB431542 inhibits TGF-β1-induced upregulation of lincRNA-p21. AML12 cells were treated by 5 ng/μl TGF-β1 for 24 h in the presence of 2 μM SB431542. LincRNA-p21 was determined by qRT-PCR. The results are shown as fold change compared with cells without TGF-β1 treatment. (B) TGF-β exhibits no effect on p53 production in hepatocyte. AML12 cells were treated by 5 ng/μl TGF-β1 for 24 h. P53 production was detected by western blot. (C) TGF-β exhibits no effect on pri-miR-30s transcription in hepatocyte. AML12 cells were treated by 5 ng/μl TGF-β1 for 24 h. pri-miR-30a, -30b, -30c, -30d and -30e were determined by qRT-PCR. The results are shown as fold change compared with cells without TGF-β1 treatment. Data are the mean ±SEM of three independent experiments. **P < 0.01. NS, no significant change.

Supplementary Figure S5. (A) LincRNA-p21 increased the luciferase activity of the reporter containing KLF11 3'UTR. AML12 cells were cotransfected with luciferase reporter containing wild or mutant KLF11 3'UTR, and pCI-lincRNA-p21. (B) miR-30b blunted the effects of lincRNA-p21 on luciferase activity. AML12 cells were cotransfected with luciferase reporter, pCI-lincRNA-p21, and miR-30b as indicated. (C) Knockdown of lincRNA-p21 decreased the luciferase activity of the reporter containing KLF11 3'UTR. AML12 cells were cotransfected with luciferase reporter, lincRNA-p21 siRNA, and miR-30 antagomir as indicated. The results are shown as fold change compared with control. Data are the mean±SEM of three independent experiments. *$P < 0.05$, **$P < 0.01$.

Supplementary Figure S6. (A) Knockdown of lincRNA-p21 inhibited TGF-β1-inuced hepatocyte apoptosis. AML12 cells were transfected with lincRNA-p21 siRNA for 48 h in the presence of TGF-β1. Cell apoptosis was determined by FACS analysis. (B) Effects of lincRNA-p21 siRNA on TGF-β1-inuced deregulation of Bax, Bad and Bcl-XL in AML12 cells. (C) Knockdown of lincRNA-p21 inhibited caspase-3 activation. Caspase-3 and cleaved caspase-3 were detected by western blot. mRNA expressions were determined by qRT-PCR. The results are shown as fold change compared with control group. Data are the mean±SEM of three independent experiments. *P < 0.05, **P < 0.01.

Supplementary Figure S7. lincRNA-p21 contributes to regulating hepatocyte growth. (A) Ectopic expression of lincRNA-p21 decreased AML12 cell viability. AML12 cells were transfected with

pCI-lincRNA-p21 for indicated times. (B) Knockdown of lincRNA-p21 increased AML12 cell viability. AML12 cells were transfected with lincRNA-p21 siRNA for indicated times. (C and D) Knockdown of lincRNA-p21 impaired (C) TGF-β1 or (D) doxorubicin-induced AML12 cell death. AML12 cells were transfected with lincRNA-p21 siRNA for indicated times in the presence of 5 ng/ml TGF-β1 or 0.8 μg/ml doxorubicin. (E) Immunohistochemical staining of liver sections from CCl4+AdH-shNC and CCl4+AdH-shlincp21 groups with antibody specific for Ki67. Original magnification 200×. Data are the mean±SEM of three independent experiments. *P < 0.05, **P < 0.01.

Supplementary Figure S8. Hypothetical roles of hepatocyte lincRNA-p21 in TGFβ signaling and liver fibrogenesis

Supplementary Figure S9. The original northern blot and gel images of Figure 1.

Supplementary Figure S10. The original immunoblot images of Figure 7.

Supplementary Figure S11. The original immunoblot images of Figure 8.

Supplementary Figure S1

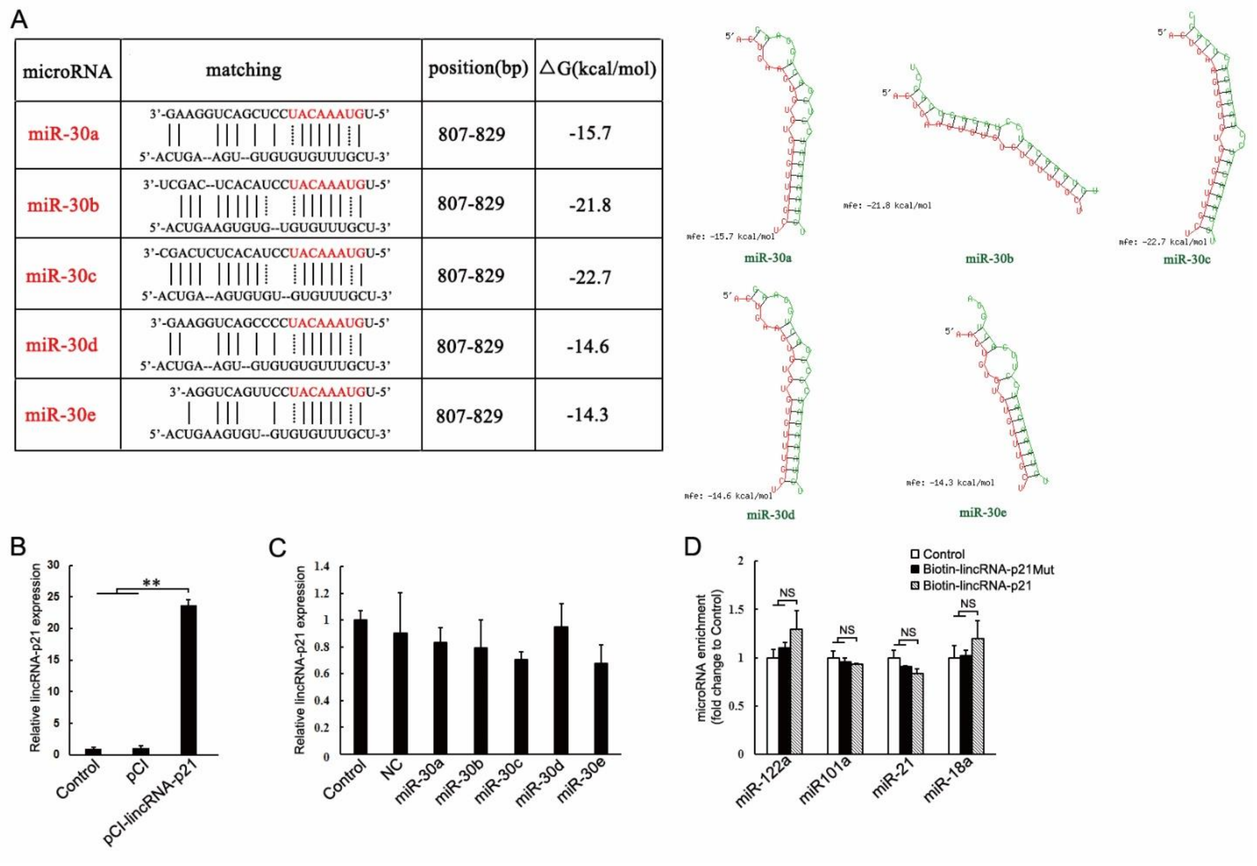

microRNA	matching	position(bp)	ΔG(kcal/mol)
miR-30a	3'-GAAGGUCAGCUCCUACAAAUGU-5'<br>5'-ACUGA--AGU--GUGUGUGUUUGCU-3'	807-829	-15.7
miR-30b	3'-UCGAC--UCACAUCCUACAAAUGU-5'<br>5'-ACUGAAGUGUG--UGUGUUUGCU-3'	807-829	-21.8
miR-30c	3'-CGACUCUCACAUCCUACAAAUGU-5'<br>5'-ACUGA--AGUGUGU--GUGUUUGCU-3'	807-829	-22.7
miR-30d	3'-GAAGGUCAGCCCCUACAAAUGU-5'<br>5'-ACUGA--AGU--GUGUGUGUUUGCU-3'	807-829	-14.6
miR-30e	3'-AGGUCAGUUCCUACAAAUGU-5'<br>5'-ACUGAAGUGU--GUGUGUUUGCU-3'	807-829	-14.3

Supplementary Figure S2

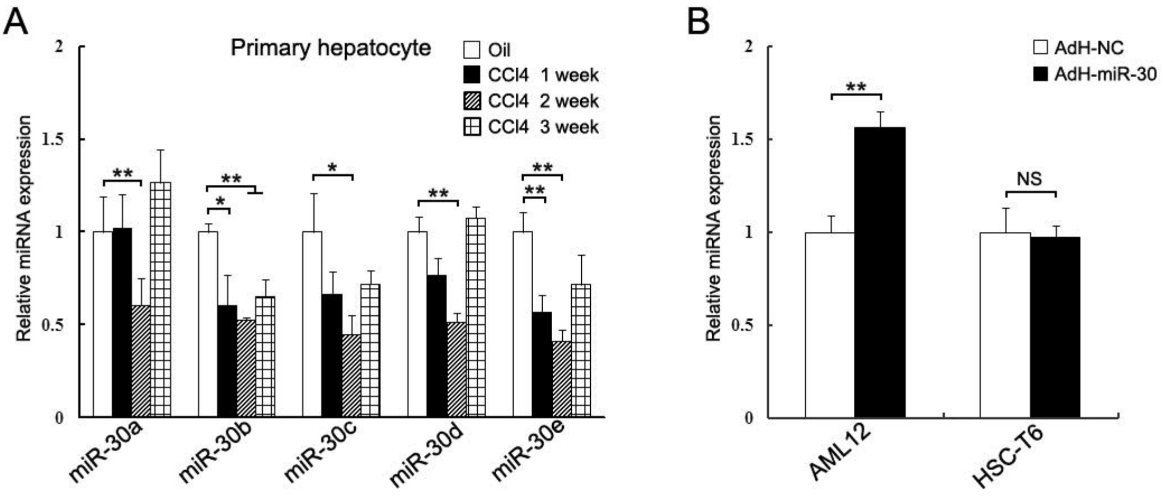

Supplementary Figure S3

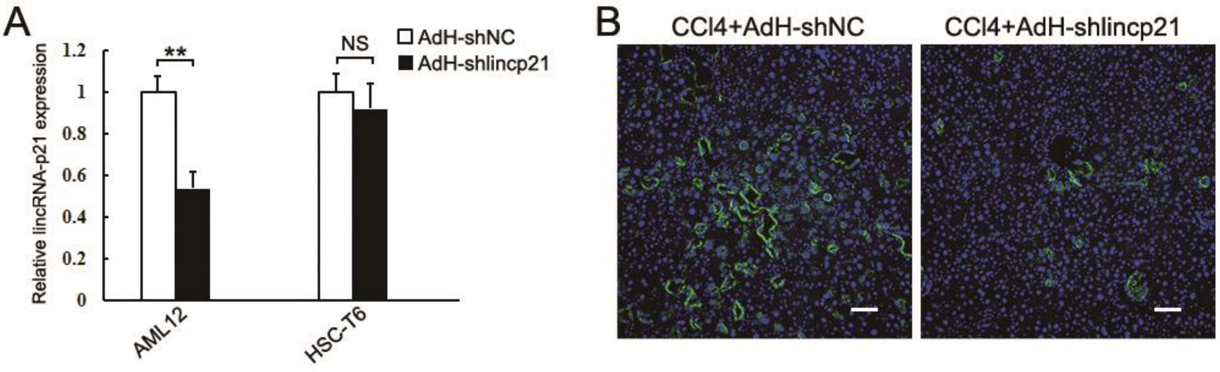

Supplementary Figure S4

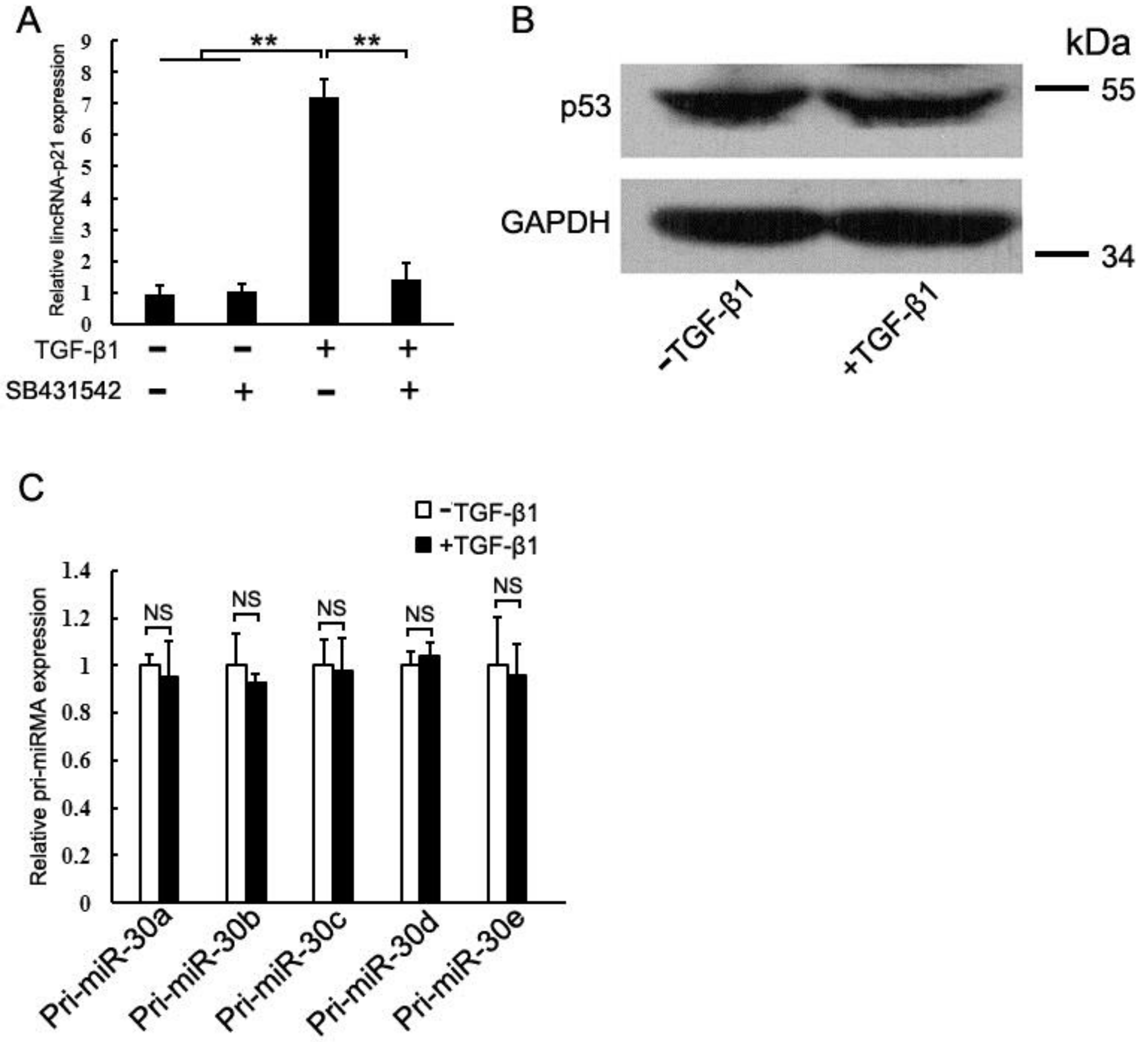

Supplementary Figure S5

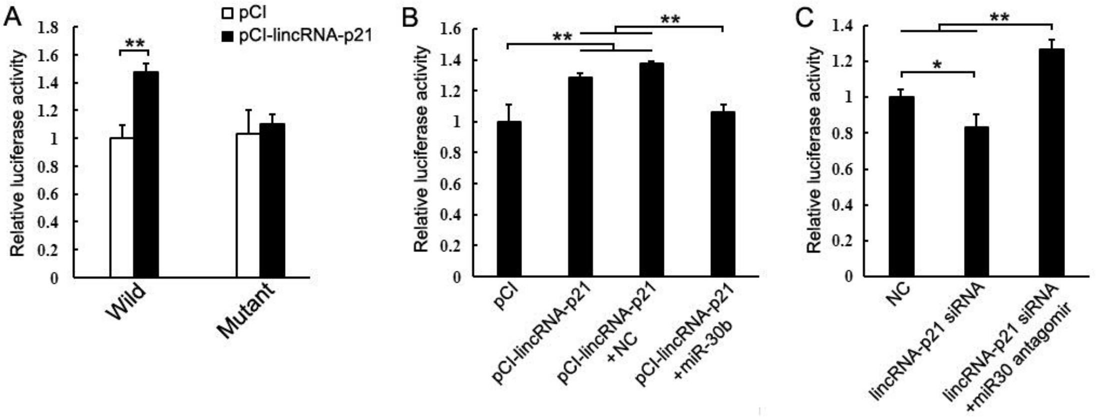

Supplementary Figure S6

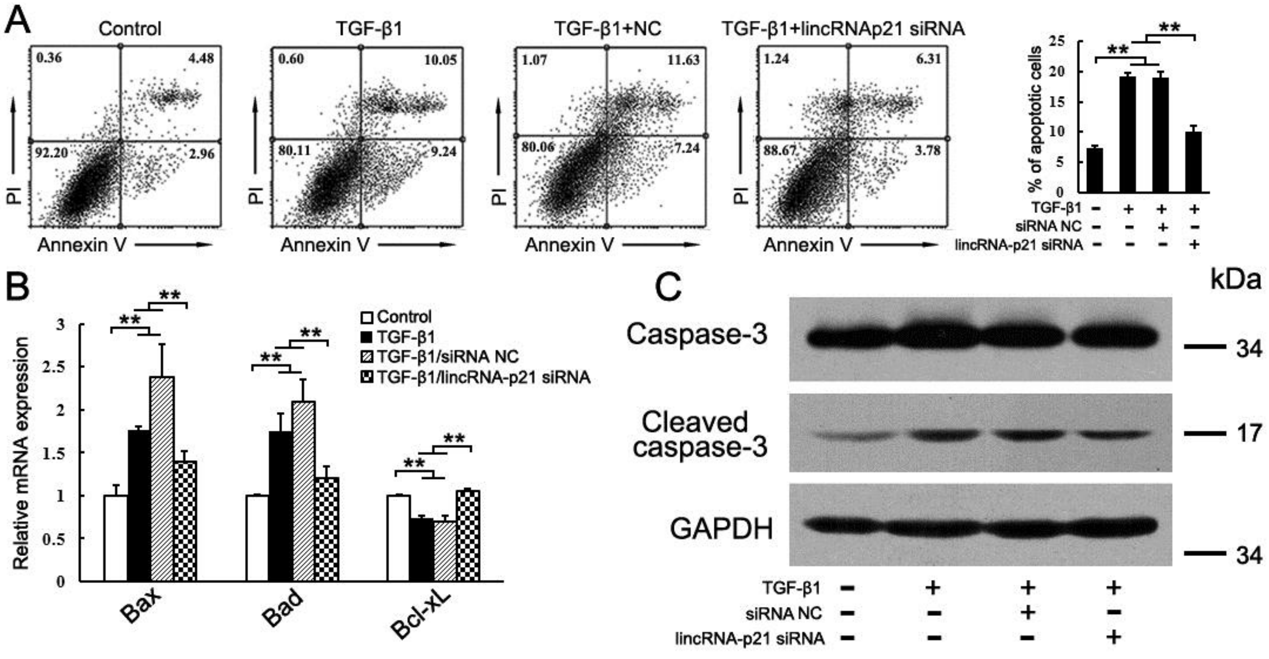

Supplementary Figure S7

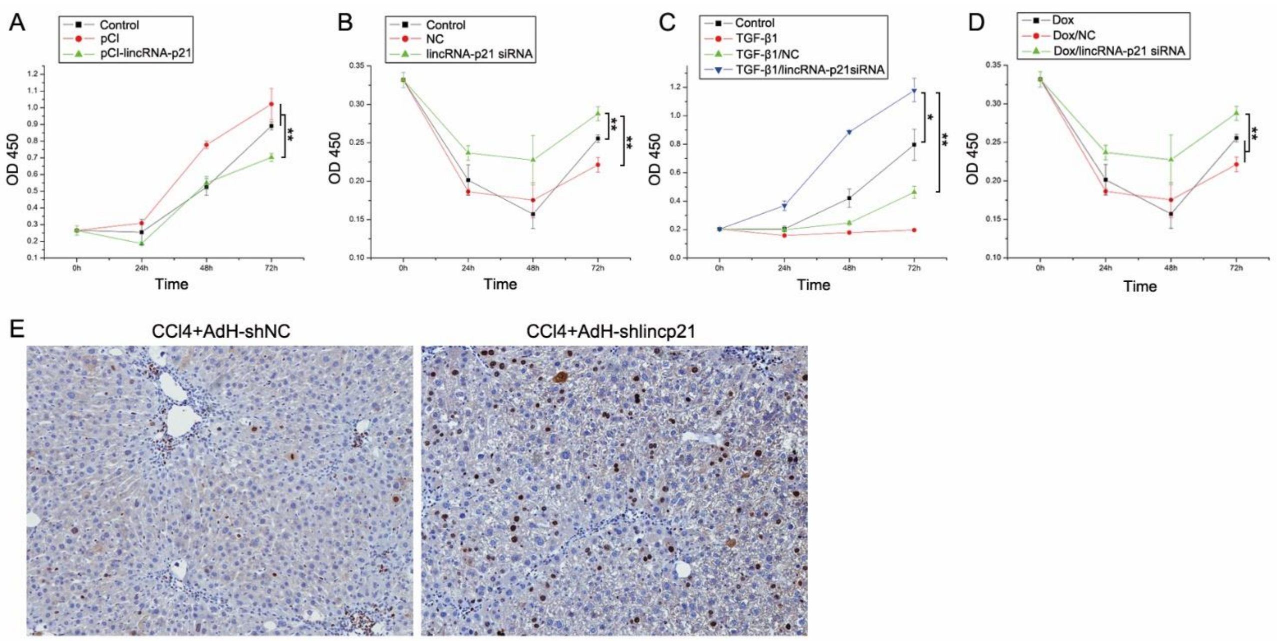

Supplementary Figure S7

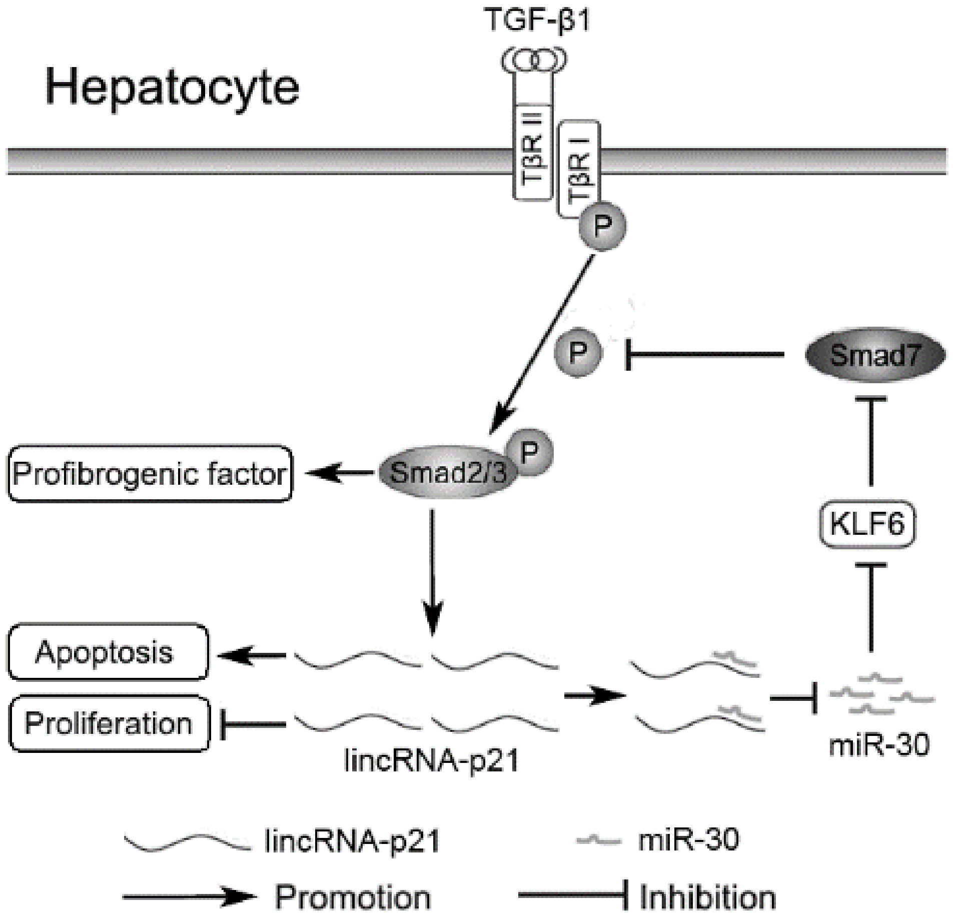

Supplementary Figure S9

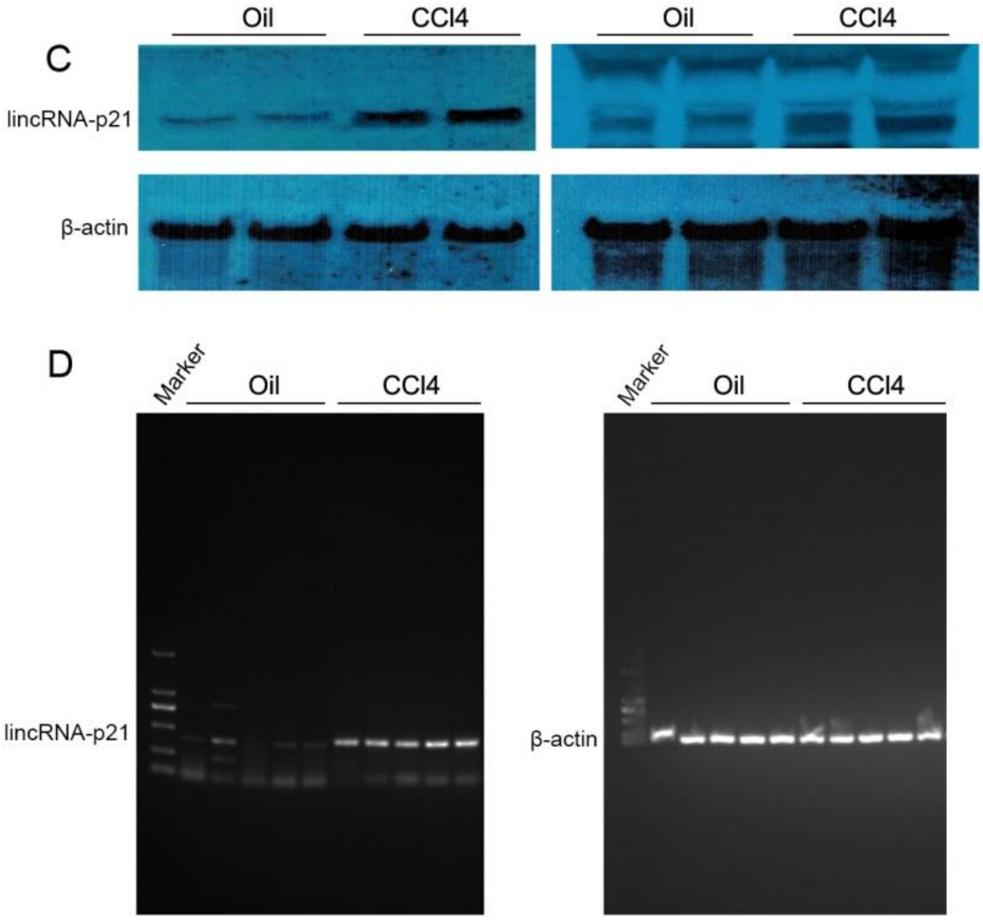

Supplementary Figure S10

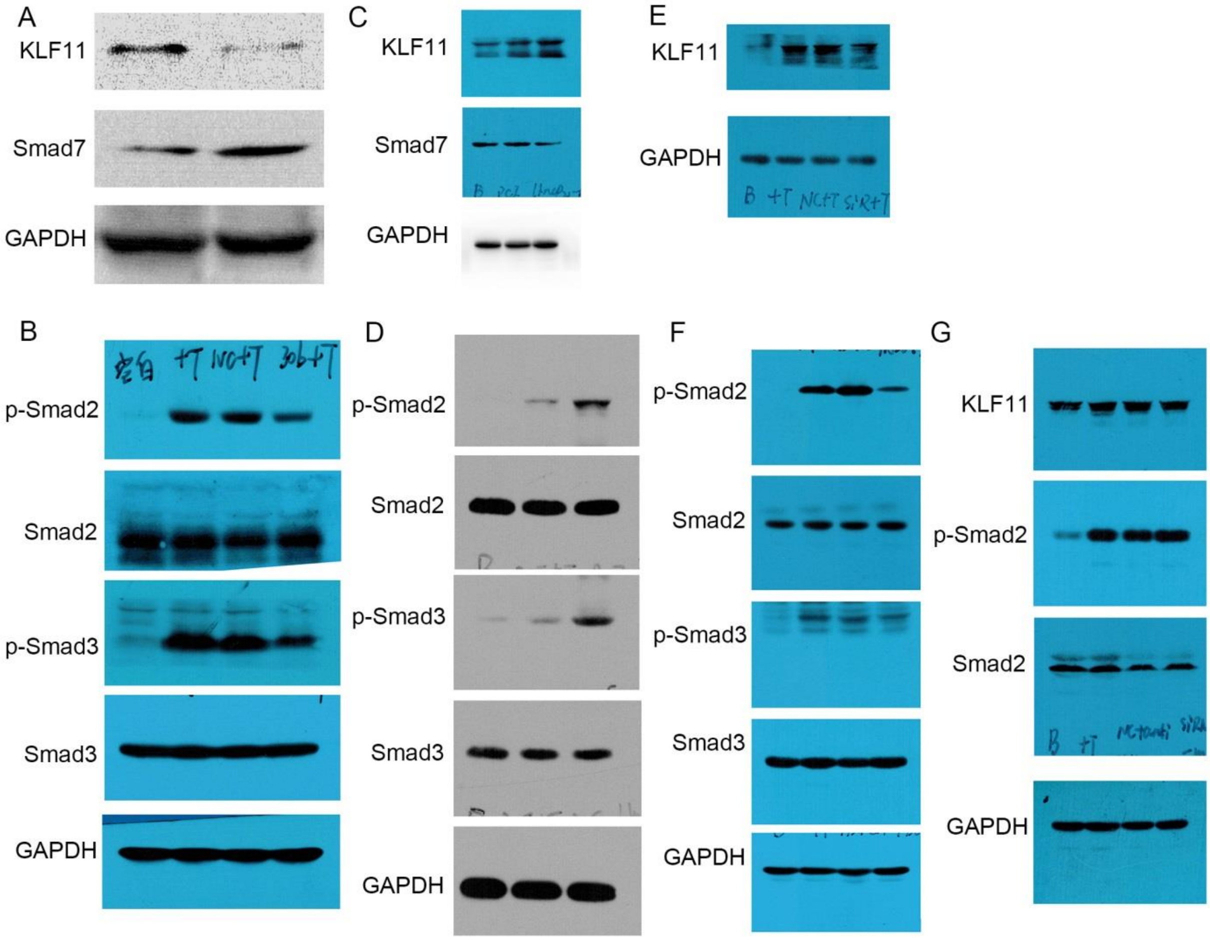

Supplementary Figure S11

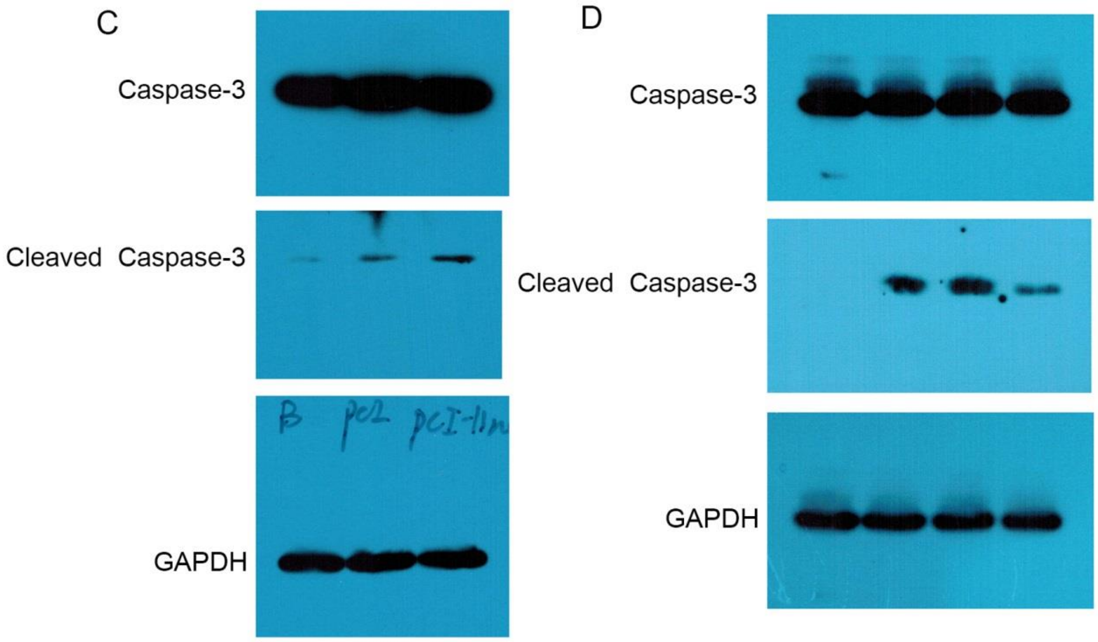

Supplement: Supplementary file 1 — Supplementary file [file 41598_2017_3175_MOESM1_ESM.pdf]
